# ZNF33B Promotes Japanese Encephalitis Virus Infection by Regulating the Stability of M^6^A‐Modified *Trim25* to Control the Autophagy Process

**DOI:** 10.1002/advs.76122

**Published:** 2026-06-18

**Authors:** Jian Du, Chunwei Li, Jiyuan Luo, Huizhi Zhang, Jinyan Zhang, Suya Wang, Huanchun Chen, Hongli Xu, Xiangmin Li, Ping Qian

**Affiliations:** ^1^ National Key Laboratory of Agricultural Microbiology, Hubei Hongshan Laboratory Huazhong Agricultural University Wuhan 430070 P. R. China; ^2^ College of Veterinary Medicine Huazhong Agricultural University Wuhan 430070 P. R. China; ^3^ The Cooperative Innovation Centre for Sustainable Pig Production Huazhong Agricultural University Wuhan 430070 P. R. China; ^4^ Hubei Cancer Hospital, Tongji Medical College, Huazhong University of Science and Technology Hubei Provincial Clinical Research Center For Colorectal Cancer Wuhan 430079 P. R. China; ^5^ Hubei Jiangxia Laboratory Wuhan 430200 P. R. China

## Abstract

Japanese encephalitis virus (JEV) is a neurotropic flavivirus that causes a substantial threat to human health and livestock; however, the epitranscriptomic mechanisms that support its replication remain poorly defined. Here, we identify a proviral host factor C_2_H_2_ zinc‐finger protein ZNF33B that promotes JEV infection through coupling N^6^‐methyladenosine (m^6^A) RNA modification to autophagy regulation. Mechanistically, ZNF33B recruits METTL14 to stabilize the METTL3‐METTL14 methyltransferase complex, thereby increasing global m^6^A deposition. Multi‐omics analyses reveal that ZNF33B selectively binds m^6^A‐modified sites within the antiviral transcript *Trim25* (c.1567 and c.1669 bp) to accelerate its decay. We further demonstrate that TRIM25 functions as an E3 ubiquitin ligase that catalyzes K48‐linked ubiquitination of ATG7 at lysines 389 and 423, leading to its proteasomal degradation and ultimately suppressing autophagic flux. In contrast, ZNF33B‐mediated *Trim25* degradation counteracts its inhibitory effect on autophagy, creating a favorable environment for viral replication. In vivo, adeno‐associated virus (AAV)‐mediated ZNF33B delivery increases mouse brain m^6^A levels, decreases TRIM25 expression, elevates ATG7 abundance, exacerbates JEV‐induced neuropathology, and accelerates mouse mortality. Together, these findings reveal a previously uncharacterized ZNF33B‐m^6^A‐TRIM25‐autophagy axis that JEV hijacks to evade host antiviral responses, providing new insights into flaviviral pathogenesis and potential therapeutic targets.

## Graphical abstract

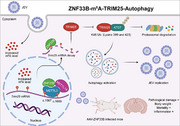

## 1 Introduction

Japanese encephalitis virus (JEV) is a mosquito‐borne flavivirus that causes severe viral encephalitis, predominantly affecting children and leading to high mortality and long‐term neurological sequelae among survivors [1, 2]. Following neuroinvasion, JEV disrupts the blood‐brain barrier (BBB) and triggers robust neuroinflammatory responses, resulting in extensive neuronal damage [3]. Despite advances in vaccine development, the complex mechanisms underlying JEV pathogenesis and host antiviral responses remain incompletely understood, highlighting the urgent need to identify key regulatory nodes in virus‐host interactions.

Zinc‐finger proteins (ZFPs) comprise a substantial family of regulatory elements characterized by zinc‐coordinating domains that mediate interactions with DNA, RNA, or proteins [4, 5]. In addition to their canonical roles as transcription factors, many C_2_H_2_‐type ZFPs function as RNA‐binding proteins that regulate post‐transcriptional processes, including RNA splicing, stability, localization, and modification [6, 7]. Several ZFPs have been implicated in virus‐host interactions by modulating host antiviral gene expression or viral RNA metabolism [8, 9]. However, the specific functions and mechanisms of most ZFPs during JEV infection remain largely unexplored.

In recent years, RNA modifications, particularly N^6^‐methyladenosine (m^6^A), have emerged as a central focus in epitranscriptomic regulation of virus‐host interactions [10, 11]. As the most prevalent internal chemical modification in eukaryotic mRNA, m^6^A is deposited by methyltransferase complexes (e.g., METTL3/METTL14/WTAP), removed by demethylases (e.g., FTO, ALKBH5), and recognized by reader proteins (e.g., YTH domain family). Through these coordinated actions, m^6^A influences RNA splicing, export, stability, and translation [12, 13, 14]. Accumulating evidence indicates that numerous RNA viruses, including human immunodeficiency virus (HIV), influenza virus (IAV), and Zika virus (ZIKV), exploit or reshape the host m^6^A landscape to optimize their replication or evade immune surveillance [15, 16, 17]. In the context of JEV, the m^6^A writer METTL3 has been reported to promote viral proliferation by altering innate immune response [18]. Nevertheless, how m^6^A modification is dynamically regulated during JEV infection, and how it intersects with specific RNA‐binding proteins, remains poorly understood.

Autophagy is a conserved catabolic pathway that delivers cytoplasmic cargo to lysosomes for degradation and plays a context‐dependent role during viral infection [19, 20]. While autophagy can function as an intrinsic antiviral defense through xenophagic clearance of viral components, many viruses, including JEV, dengue virus (DENV), and hepatitis C virus (HCV), have evolved to subvert the autophagy pathway, utilizing autophagic membranes as scaffolds for replication complexes or to dampen antiviral signaling [21, 22, 23]. Core autophagy machinery proteins (e.g., ATG5, ATG7, and LC3) are tightly regulated at transcriptional and post‐translational levels [24, 25]. Notably, members of the tripartite motif (TRIM) family of E3 ubiquitin ligases have emerged as important modulators of autophagy through ubiquitination of autophagy‐related proteins [26, 27]. TRIM25 is a well‐characterized antiviral protein known primarily for its role in catalyzing K63‐linked ubiquitination of RIG‐I to activate type I interferon signaling [28]. However, whether and how TRIM25 regulates autophagy to impact JEV replication is unknown.

Here, we identified that the C_2_H_2_ zinc‐finger protein (Cys2‐His2 zinc finger proteins, C_2_H_2_ ZNFs) ZNF33B is a critical host factor co‐opted by JEV to integrate m^6^A RNA modification with autophagy regulation. We demonstrate that ZNF33B recruits METTL14 to enhance m^6^A deposition and selectively targets the antiviral transcript *Trim25* for m^6^A‐dependent decay. By downregulating TRIM25, ZNF33B relieves its inhibitory effect on autophagy by preventing K48‐linked ubiquitination and degradation of ATG7, thereby promoting autophagic flux and viral replication. Using both in vitro and in vivo models, we establish a ZNF33B‐m^6^A‐TRIM25‐ATG7 axis that is hijacked by JEV to evade host antiviral defenses. These findings provide mechanistic insight into flaviviral pathogenesis and nominate epitranscriptomic‐autophagic crosstalk as a potential target for host‐directed antiviral therapy.

## 2 Results

### 2.1 ZNF33B Stabilizes the M^6^A Writer Complex by Recruiting METTL14 to Promote JEV Replication

Our prior research indicated that ZNF33B facilitates JEV replication by integrating m^6^A modification and RNA metabolism to elude host immunity [29]. Hence, we initially examined whether JEV infection alters cellular m^6^A modification. An immunofluorescence assay using an antibody capable of recognizing m^6^A‐modified nucleic acids revealed that m^6^A signals accumulated within the cytoplasm during JEV infection (Figure S1A,B). Subsequently, the impact of JEV infection on the levels of m^6^A writers was determined. The results showed that JEV infection increased the protein abundance of the m^6^A writer components METTL3 and METTL14 (Figure S1C,D,E), suggesting that m^6^A deposition is dynamically regulated during infection.

**FIGURE 1:**
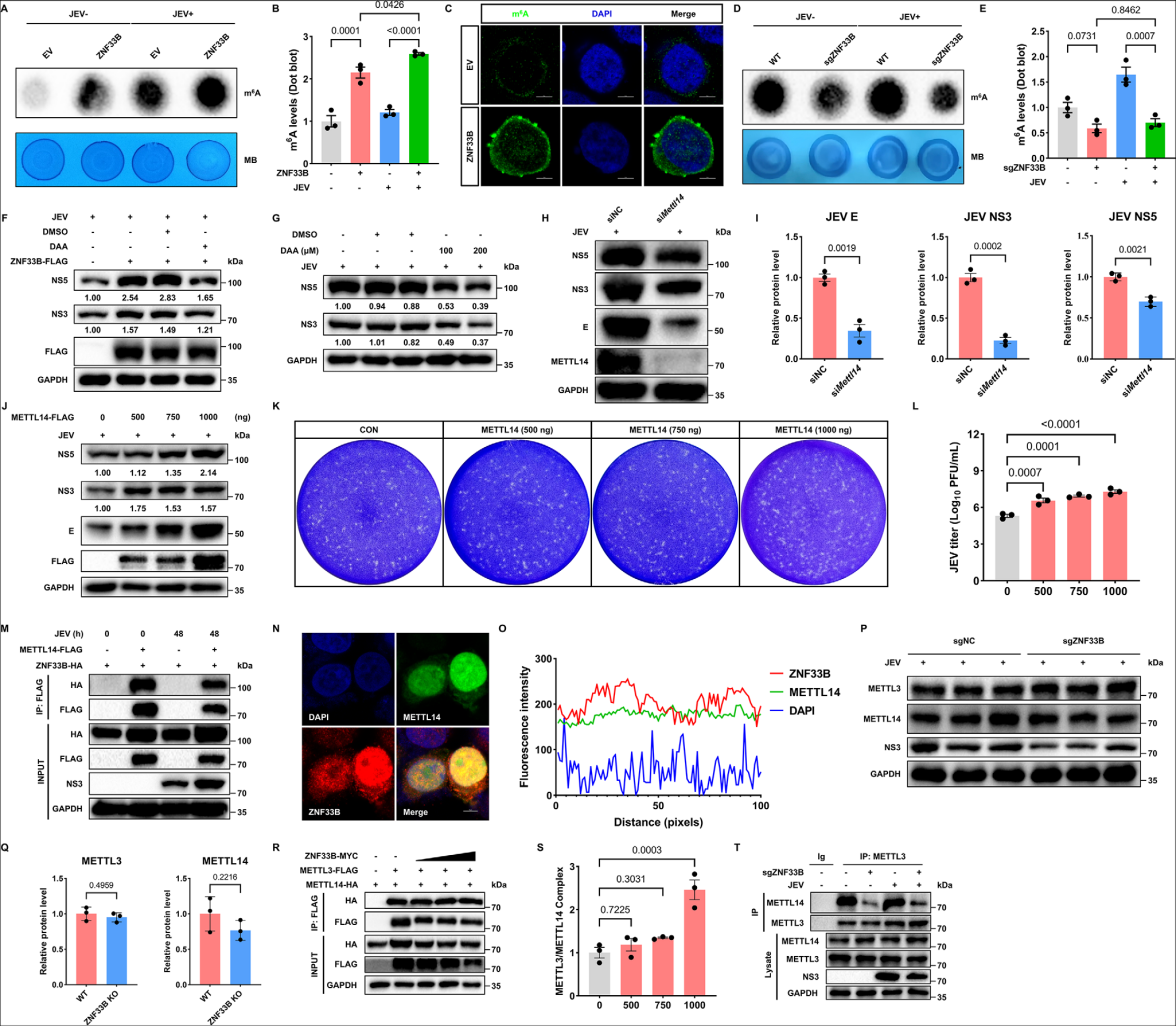
ZNF33B stabilizes the m^6^A writer complex by recruiting METTL14 to promote JEV replication. (A) RNA dot blot analysis of m6A levels in HEK293T cells transfected with empty vector control or ZNF33B‐FLAG, followed by JEV for 48 h. Methylene blue staining served as a loading control. (B) The levels of m^6^A were assessed by measuring the band grayscale with the “ImageJ” software. (C) The confocal microscope observation on m^6^A signal in HEK293T cells transfected with empty vector control or ZNF33B‐FLAG, followed by JEV for 48 h. Scale bar: 5 µm. (D) RNA dot blot analysis of m^6^A levels in PK15 WT and ZNF33B KO cells infected by JEV for 48 h. Methylene blue staining served as a loading control. (E) The levels of m^6^A were assessed by measuring the band grayscale with the “ImageJ” software. (F) Immunoblot analysis of the expression of JEV NS3 and NS5 in JEV‐infected HEK293T cells transfected with empty vector control or ZNF33B‐FLAG, followed by DMSO or methylation inhibitor 3‐Deazaadenosine (DAA) treatment for 12 h. The expressions of JEV NS3 and NS5 were assessed by measuring the band grayscale with the “ImageJ” software. (G) Immunoblot analysis of the expression of JEV NS3 and NS5 in JEV‐infected HEK293T cells, followed by DMSO or DAA (100, 200 µM) treatment for 12 h. The expressions of JEV NS3 and NS5 were assessed by measuring the band grayscale with the “ImageJ” software. (H) Immunoblot analysis of the expression of JEV E, NS3, NS5, and METTL14 in HEK293T cells transfected with negative control or METTL14 siRNAs, followed by JEV for 48 h. (I) The expressions of JEV E, NS3, and NS5 were assessed by measuring the band grayscale with the “ImageJ” software. (J) Immunoblot analysis of the expression of JEV NS3 and NS5 in HEK293T cells transfected with empty vector control or METTL14‐FLAG, followed by JEV for 48 h. The expressions of JEV NS3 and NS5 were assessed by measuring the band grayscale with the “ImageJ” software. (K) The viral titration analysis of the supernatant in JEV‐infected cells expressing METTL14 was conducted by plaque assay. (L) The statistical analysis of JEV titer in METL14‐overexpressed cells. (M) Immunoblot analysis of the association of ZNF33B with METTL14 by immunoprecipitation of lysates from HEK293T cells transfected with ZNF33B‐HA and METTL14‐FLAG, followed by JEV infection for 48 h. The cell lysates were immunoprecipitated with anti‐FLAG antibodies. (N) Confocal microscope observation of the colocalization of ZNF33B with METTL14 in HEK293T cells co‐transfected with ZNF33B‐HA and METTL14‐FLAG, followed by JEV infection for 48 h. Scale bar: 5 µm. (O) The colocalization of ZNF33B with METTL14 was quantitatively assessed by measuring the fluorescence intensity with the “ImageJ” software. (P) Immunoblot analysis of the expression of METTL3 and METTL14 in PK15 WT and ZNF33B KO cells infected by JEV for 48 h. (Q) The expression of METTL3 and METTL14 was assessed by measuring the band grayscale with the “ImageJ” software. (R) Immunoblot analysis of the association of METTL3 with METTL14 by immunoprecipitation of lysates from HEK293T cells transfected with METTL3‐FLAG and METTL14‐HA in combination with ZNF33B‐MYC, followed by JEV infection for 48 h. The cell lysates were immunoprecipitated with anti‐FLAG antibodies. (S) The levels of METTL3/METTL14 complex were assessed by measuring the band grayscale with the “ImageJ” software. (T) Immunoblot analysis of the association of METTL3 with METTL14 by immunoprecipitation of lysates from PK15 WT and ZNF33B KO cells infected by JEV for 48 h. The cell lysates were immunoprecipitated with anti‐METTL3 antibodies. All experiments were conducted in triplicate, and data are represented as mean ± SD. Statistical analysis was performed by a two‐tailed Student's *t*‐test (I and Q) or one‐way ANOVA with Tukey's multiple comparisons (B, E, L, and S).

To determine whether ZNF33B influences m^6^A modification, we performed m^6^A dot blot assays following ZNF33B overexpression or depletion. The results demonstrated that m^6^A level increased by ZNF33B overexpression regardless of JEV infection (Figure 1A,B). Moreover, we observed that ZNF33B enhanced the accumulation of m^6^A signals in the cytoplasm (Figure 1C). Conversely, knockout of ZNF33B markedly reduced the m^6^A level (Figure 1D,E). To further investigate whether JEV replication promoted by ZNF33B is dependent on the hyper‐m^6^A level, ZNF33B‐overexpressing HEK293T cells were infected with JEV for 36 h. Subsequently, the cells were treated with DMSO or methylation inhibitor 3‐Deazaadenosine (DAA) for 12 h. The results revealed a significant enhancement in the abundances of JEV NS3 and NS5 proteins under overexpression of ZNF33B. However, DAA treatment abrogated the enhancement of JEV NS3 and NS5 proteins (Figure 1F). Similarly, knockdown of methyltransferase METTL3 or overexpression of demethylase FTO attenuated the advantage of ZNF33B in JEV replication (Figure S2A–C). Consistent with these findings, inhibition of m^6^A methylation by DAA alone reduced the expression of JEV NS3 and NS5 proteins (Figure 1G). Collectively, these data demonstrate that ZNF33B promotes JEV replication in a manner that requires cellular hyper‐m^6^A modification.

To investigate how ZNF33B enhances m^6^A deposition, we systematically examined its binding partners by co‐transfecting HEK293T cells with HA‐tagged ZNF33B and FLAG‐tagged expression plasmids encoding METTL3, METTL14, WTAP, YTHDF1, YTHDF2, YTHDF3, and FTO. Co‐immunoprecipitation analyses revealed that ZNF33B selectively interacts with METTL14, WTAP, YTHDF2, and YTHDF3, but not with METTL3, YTHDF1, or FTO (Figure S3A–G). Similarly, we observed that ZNF33B could interact with endogenous METTL14, WATP, YTHDF2, and YTHDF3 (Figure S3H). Given the central role of METTL14 in stabilizing the METTL3‐METTL14 catalytic core, we focused on its functional relevance during JEV infection. As anticipated, we observed an impaired capacity of JEV replication in METTL14 knockdown cells (Figure 1H,I). In contrast, METTL14 overexpression markedly increased the expression of JEV E, NS3, and NS5 proteins (Figure 1J). Furthermore, at 48 h post‐infection (hpi), the viral titer from infected cell supernatant was measured by plaque assay. Significantly, an elevation in viral loads was detected in METTL14‐overexpressing cells (Figure 1K,L). Since both ZNF33B and METTL14 positively regulate JEV replication, transient transfection and co‐immunoprecipitation experiments were conducted to validate the interaction between METTL14 and ZNF33B in the presence of JEV infection. The results demonstrated that ZNF33B was associated with METTL14 irrespective of JEV infection (Figure 1M). Moreover, IFA results indicated that ZNF33B was predominantly co‐localized with METTL14 in the nucleus (Figure 1N,O). Therefore, we hypothesized that ZNF33B might regulate JEV replication by modulating the activity of METTL3 and METTL14. First, we assessed the effect of ZNF33B on the levels of these two m^6^A methyltransferases. Surprisingly, the results revealed that ZNF33B did not alter the protein levels of METTL3 or METTL14 (Figure 1P,Q). In the process of m^6^A deposition on mRNAs, METTL3 serves as the primary catalytic subunit, whereas METTL14 is catalytically inactive but stabilizes the RNA‐binding ability of METTL3. Together, these two proteins form a stable METTL3‐METTL14 heterodimeric core complex that mediates m^6^A methylation of nuclear RNAs in mammalian cells. Hence, we further detected the interaction of METTL3 and METTL14 in MYC‐tag fused ZNF33B‐overexpressed cells. We observed that the METTL3‐METTL14 heterodimer was significantly increased in ZNF33B‐expressed cells (Figure 1R,S). Conversely, knockout of ZNF33B impaired the association between METTL3 and METTL14, indicating that ZNF33B may adjust the RNA methylation modification activity of the m^6^A writer complex (Figure 1T). Taken together, our findings suggested that ZNF33B promotes m^6^A deposition by recruiting METTL14 and potentiating the activity of the m^6^A methyltransferase complex, rather than by altering writer expression, to promote JEV replication.

### 2.2 Multi‐Omics Analyses Identify *Trim25* as a Dominant ZNF33B‐Associated Antiviral Transcript

**FIGURE 2:**
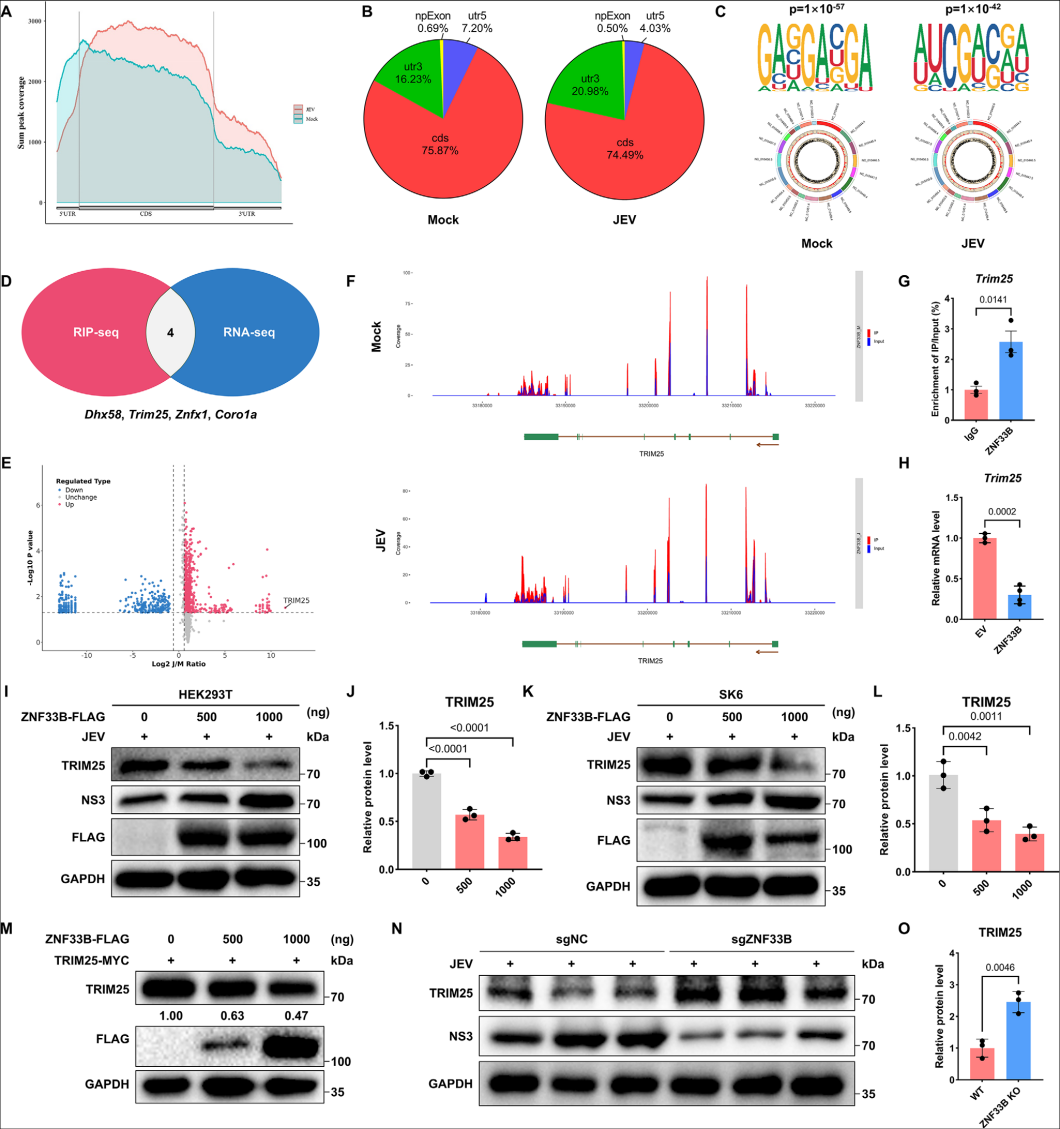
Multi‐omics analyses identify *Trim25* as a dominant ZNF33B‐associated antiviral transcript. (A) Metagene profiles illustrating the distribution of sum RIP peaks along transcripts by exomePeak in Mock or JEV‐infected SK6 cells, categorized into three segments: 5’UTR, CDS, and 3’UTR. (B) Distribution of RIP peaks across the genome, presented as a pie chart. cds refers to the CDS region of the gene, while utr5 and utr3 are the 5’UTR and 3’UTR of the gene, respectively. npExon is the exon region of non‐coding genes. (C) Identification of peaks in chromosome distribution and enriched motifs within peaks from RIP‐seq data. (D) The intersection of data derived from the RIP‐seq data and RINA‐seq data, with 4 overlapping ZNF33B‐associated genes being presented. (E) A volcano plot depicting differentially expressed peak genes, with upregulated genes shown in pink and downregulated genes in blue by comparing the JEV and Mock‐infected group. (F) Visualization of RIP‐seq read coverage on *Trim25* mRNA using Integrative Genomics Viewer (IGV). (G) RNA immunoprecipitation (RIP) assay using anti‐HA antibody and qPCR analysis of the association between ZNF33B protein and *Trim25* mRNA with specific primers in HEK293T cells transfected with HA‐tagged ZNF33B, followed by JEV infection for 48 h. (H) qPCR analysis of the level of *Trim25* mRNA with specific primers in HEK293T cells transfected with ZNF33B‐HA, followed by JEV infection for 48 h. (I) Immunoblot analysis of the expression of TRIM25 in HEK293T cells transfected with empty vector control or ZNF33B‐FLAG, followed by JEV for 48 h. (J) The expression of TRIM25 was assessed by measuring the band grayscale with the “ImageJ” software. (K) Immunoblot analysis of the expression of TRIM25 in SK6 cells transfected with empty vector control or ZNF33B‐FLAG, followed by JEV for 48 h. (L) The expression of TRIM25 was assessed by measuring the band grayscale with the “ImageJ” software. (M) Immunoblot analysis of the expression of TRIM25 in HEK293T cells transfected with TRIM25‐MYC in combination with empty vector control or ZNF33B‐FLAG, followed by JEV for 48 h. (N) Immunoblot analysis of the expression of TRIM25 in PK15 WT and ZNF33B KO cells infected by JEV for 48 h. (O) The expression of TRIM25 was assessed by measuring the band grayscale with the “ImageJ” software. All experiments were conducted in triplicate, and data are represented as mean ± SD. Statistical analysis was performed by a two‐tailed Student's *t*‐test (G, H, and O) or one‐way ANOVA with Tukey's multiple comparisons (J and L).

Accumulating evidence has demonstrated that C_2_H_2_ zinc finger proteins are capable of binding to RNA and regulating multiple post‐transcriptional processes, including precursor mRNA splicing, mRNA degradation, polyadenylation, and m^6^A modification [6, 7]. Furthermore, our previous findings have indicated that ZNF33B facilitates JEV replication by mediating m^6^A modification of antiviral transcripts, implying that ZNF33B may integrate m^6^A modification to regulate RNA fate [29]. Accordingly, we performed RNA immunoprecipitation (RIP)‐seq and RNA‐seq (RIP input) assays to identify ZNF33B‐associated mRNAs in JEV‐infected cells relative to uninfected control cells. RIP‐seq data revealed that the ZNF33B preferred motifs were predominantly located in the regulatory sequence, especially the coding sequence (CDS) region (more than half), and JEV infection promoted the distribution of RIP peaks in the CDS and 3’UTR region (Figure 2A,B). After peak calling, we identified a highly enriched “RGAC” consensus motif in Mock and JEV‐infected cells consistent with the canonical m^6^A DRACH sequence (Figure 2C). Moreover, a comparison of the abundance of differentially expressed genes (DEGs) between Mock and JEV‐infected cells showed that a total of 453 DEGs were identified as significant candidates (Figure S4A). Following peak calling and annotation, the enrichment of DEGs was primarily categorized into processes related to antiviral responses, including the type I interferon signaling pathway, NOD‐like receptor signaling pathway, and RIG‐I‐like receptor signaling pathway (Figure S4B,C). Additionally, analysis of mRNA alternative splicing events identified 1524 differential alternative splicing events, and these identified events were enriched in pathways related to autophagy, RNA binding, and chromatin binding (Figure S4D–F).

To clarify the relationship between mRNA binding and expression mediated by ZNF33B, RNA‐seq and RIP‐seq results were intersected to identify overlapping genes. The potential target genes of ZNF33B were found to be *Dhx58*, *Trim25*, *Znfx1*, and *Coro1a* (Figure 2D,E). To verify the association of ZNF33B with these RNAs, ZNF33B‐overexpressing SK6 cells were infected with JEV for 48 h. Subsequently, cell lysates were incubated with anti‐FLAG antibody and normal IgG (as a negative control). The immunoprecipitated complexes were washed, and total RNA was extracted using Trizol reagent for subsequent RIP‐qPCR analysis. The results indicated that the antiviral transcripts *Dhx58*, *Trim25*, and *Znfx1* could be immunoprecipitated by ZNF33B protein (Figure S5A,B). Furthermore, qPCR results demonstrated that ZNF33B exerted a strong inhibitory effect on the expression of *Dhx58*, *Trim25*, and *Znfx1*. Additionally, ZNF33B was found to promote the expression of *Coro1a*, which is a key effector of autophagy (Figure S5C). Among these identified target genes, ZNF33B exhibited stronger binding affinity and inhibitory capacity toward *Trim25*. Therefore, we focused on investigating the role of TRIM25 in ZNF33B‐assisted JEV replication in subsequent experiments.

Notably, analysis using the Integrative Genomics Viewer (IGV) highlighted specific sites of ZNF33B binding on *Dhx58*, *Trim25*, *Znfx1*, and *Coro1a* mRNA, which showed a parallel region and significant abundance alteration in coding sequence and 3’UTR (Figure 2F and Figure S6A–C). Moreover, we observed that ZNF33B exhibited pronounced binding affinity and inhibitory capacity toward *Trim25* mRNA by transfecting HEK293T cells with ZNF33B‐HA plasmids during JEV infection (Figure 2G,H). Subsequently, we assessed the impact of ZNF33B on the abundance of TRIM25 protein. Upon ZNF33B overexpression, the protein level of endogenous TRIM25 declined remarkably (Figure 2I–L). Similarly, ZNF33B impaired the exogenous TRIM25 protein level (Figure 2M). In contrast, depletion of ZNF33B enhanced the endogenous TRIM25 protein level (Figure 2N,O). Collectively, these findings demonstrate that ZNF33B, as an m^6^A‐associated RNA‐binding protein, selectively binds to and suppresses antiviral transcripts, particularly *Trim25*, thereby facilitating JEV replication.

### 2.3 ZNF33B Selectively Binds m^6^A‐Modified *Trim25* mRNA to Promote Its Decay

**FIGURE 3:**
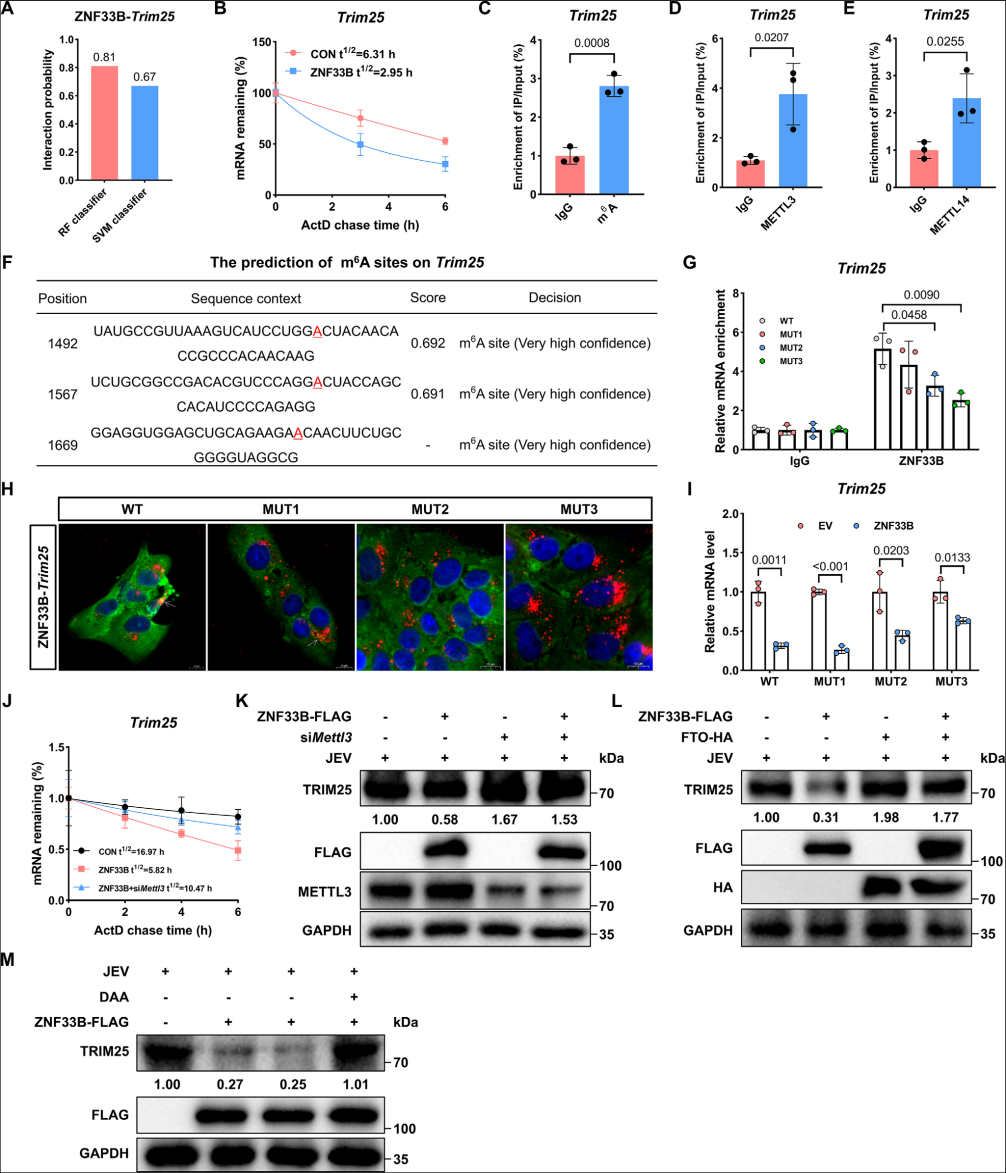
ZNF33B selectively binds m^6^A‐modified *Trim25* mRNA to promote its decay. (A) In silico prediction of ZNF33B‐*Trim25* interaction probability on the RPISeq website. (B) qPCR analysis of the level of *Trim25* mRNA in HEK293T cells transfected with ZNF33B‐HA, followed by treatment with actinomycin D (ActD, 5 µg/mL) for the indicated times. The half‐life was calculated by nonlinear regression. (C) MeRIP assay using anti‐m^6^A antibody and qPCR analysis of the m^6^A modification of *Trim25* mRNA with specific primers in HEK293T cells infected by JEV for 48 h. (D) RIP assay using anti‐HA antibody and qPCR analysis of the association between METTL3 protein and *Trim25* mRNA with specific primers in HEK293T cells transfected with HA‐tagged METTL3, followed by JEV infection for 48 h. (E) RIP assay using anti‐HA antibody and qPCR analysis of the association between METTL14 protein and *Trim25* mRNA with specific primers in HEK293T cells transfected with HA‐tagged METTL14, followed by JEV infection for 48 h. (F) The prediction of m^6^A sites on *Trim25* mRNA via the SRAMP tool (http://www.cuilab.cn/sramp/). (G) RIP assay using anti‐HA antibody and qPCR analysis of the association between ZNF33B protein and *Trim25* mRNA with specific primers in HEK293T cells transfected with HA‐tagged ZNF33B in combination with FLAG‐tagged TRIM25 WT, MUT1, MUT2, and MUT3, followed by JEV infection for 48 h. (H) FISH analysis of the colocalization of ZNF33B protein with *Trim25* mRNA in HEK293T cells co‐transfected with HA‐tagged ZNF33B in combination with FLAG‐tagged TRIM25 WT, MUT1, MUT2, and MUT3, followed by JEV infection for 48 h. Scale bar: 10 µm. (I) qPCR analysis of the level of *Trim25* mRNA with specific primers in HEK293T cells co‐transfected with HA‐tagged ZNF33B in combination with FLAG‐tagged TRIM25 WT, MUT1, MUT2, and MUT3, followed by JEV infection for 48 h. (J) qPCR analysis of the level of *Trim25* mRNA in HEK293T cells transfected with ZNF33B‐FLAG and METTL3 siRNA, followed by treatment with ActD for the indicated times. The half‐life was calculated by nonlinear regression. (K) Immunoblot analysis of the expression of TRIM25 in HEK293T cells transfected with ZNF33B‐FLAG and METTL3 siRNA, followed by JEV for 48 h. The expression of TRIM25 was assessed by measuring the band grayscale with the “ImageJ” software. (L) Immunoblot analysis of the expression of TRIM25 in HEK293T cells transfected with ZNF33B‐FLAG and FTO‐HA, followed by JEV for 48 h. The expression of TRIM25 was assessed by measuring the band grayscale with the “ImageJ” software. (M) Immunoblot analysis of the expression of TRIM25 in JEV‐infected HEK293T cells transfected with empty vector control or ZNF33B‐FLAG, followed by DMSO or DAA treatment for 12 h. The expression of TRIM25 was assessed by measuring the band grayscale with the “ImageJ” software. All experiments were conducted in triplicate, and data are represented as mean ± SD. Statistical analysis was performed by a two‐tailed Student's *t*‐test (C, D, E, and I) or one‐way ANOVA with Tukey's multiple comparisons (G).

Given that RIP‐seq and RIP‐qPCR results confirmed the interaction between ZNF33B and *Trim25* mRNA, and TRIM25 is a well‐documented antiviral protein that exerts inhibitory effects on multiple RNA viruses, we next sought to explore whether ZNF33B regulates TRIM25 function through m^6^A modification to affect JEV replication. First, the ZNF33B protein sequence and the *Trim25* mRNA sequence were submitted to the RPISeq website for in silico prediction of their interaction probability. The interaction probabilities of the ZNF33B‐*Trim25* complex predicted by the random forest (RF) classifier and support vector machine (SVM) classifier were 0.81 and 0.67, respectively, indicating a high probability of interaction between ZNF33B and *Trim25* mRNA (Figure 3A). Since our previous study has demonstrated that ZNF33B facilitates the decay of antiviral transcripts, we next investigated the effect of ZNF33B on *Trim25* mRNA stability. HEK293T cells were transfected with ZNF33BHA plasmid or empty vector (as a negative control), followed by treatment with actinomycin D (ActD) to block RNA transcription. Total RNA was harvested at different time points (0, 2, 4, and 6 h) post‐ActD treatment, and the half‐life of *Trim25* mRNA was determined by qPCR. The results indicated that ZNF33B significantly shortened the half‐life of *Trim25* mRNA (Figure 3B). Considering the critical role of the m^6^A epitranscriptome in regulating RNA metabolism, we examined the m^6^A modification level of *Trim25* mRNA in JEV‐infected HEK293T cells via MeRIP‐qPCR assay. Notably, *Trim25* mRNA was significantly enriched by the anti‐m^6^A antibody in JEV‐infected HEK293T cells (Figure 3C). Consistently, we found that *Trim25* mRNA could be significantly enriched by the methyltransferase METTL3 and METTL14 (Figure 3D,E). Subsequently, we determined the effect of m^6^A modification on the protein level of TRIM25. The data showed that overexpression of METTL3 or METTL14 significantly suppressed the level of TRIM25 protein (Figure S7A,B)

To predict m^6^A modification sites within *Trim25* mRNA sequences, the SRAMP tool (http://www.cuilab.cn/sramp/) was employed. Notably, m^6^A modification sites were identified with very high confidence at positions c. 1492 bp, c. 1567 bp, and c. 1669 bp within the *Trim25* mRNA (Figure 3F and Figure S7C). To decipher whether the ZNF33B‐mediated *Trim25* mRNA degradation process is dependent on the m^6^A modification of *Trim25* mRNA, we constructed wild‐type (WT) and mutant *Trim25* expression plasmids. Specifically, the predicted m^6^A‐modified adenine (A) residues were substituted with guanine (G) to abrogate m^6^A modification. RIP‐qPCR analysis revealed that ZNF33B could bind to *Trim25* WT and the MUT1 mRNA, but weakly associated with the MUT2 and MUT3 variants (Figure 3G). These results imply that ZNF33B specifically targets the m^6^A modification sites on *Trim25* mRNA at positions c. 1567 bp and c. 1669 bp. Additionally, we observed that the MUT2 and MUT3 constructs reduced the association of *Trim25* mRNA, as indicated by fluorescence in situ hybridization (FISH) in cells transfected with ZNF33B‐HA (Figure 3H). Correspondingly, we noticed that ZNF33B decreased mRNA levels of both *Trim25* WT and MUT1, whereas the reduction was attenuated in cells transfected with *Trim25* MUT2 or MUT3 plasmids (Figure 3I). Furthermore, knockdown of METTL3 reversed the ZNF33B‐induced reduction in *Trim25* mRNA stability (Figure 3J), suggesting that ZNF33B accelerates *Trim25* mRNA degradation through m^6^A modification. Consistently, METTL3 deficiency or overexpression of m^6^A demethylase FTO abolished the inhibition of TRIM25 protein induced by ZNF33B (Figure 3K,L). We further investigate whether the reduction of TRIM25 protein induced by ZNF33B is dependent on the hyper‐m^6^A level. The results revealed a significant reduction in the protein level of TRIM25 with ZNF33B overexpression, while the inhibition of TRIM25 protein by ZNF33B was attenuated by DAA treatment (Figure 3M). Collectively, these findings indicate that ZNF33B specifically recognizes and binds to m^6^A‐modified sites within the *Trim25* mRNA, thereby accelerating its degradation in an m^6^A‐dependent manner to suppress TRIM25 protein expression and facilitate viral evasion.

### 2.4 TRIM25 Inhibits JEV Replication by Suppressing Autophagic Flux

**FIGURE 4:**
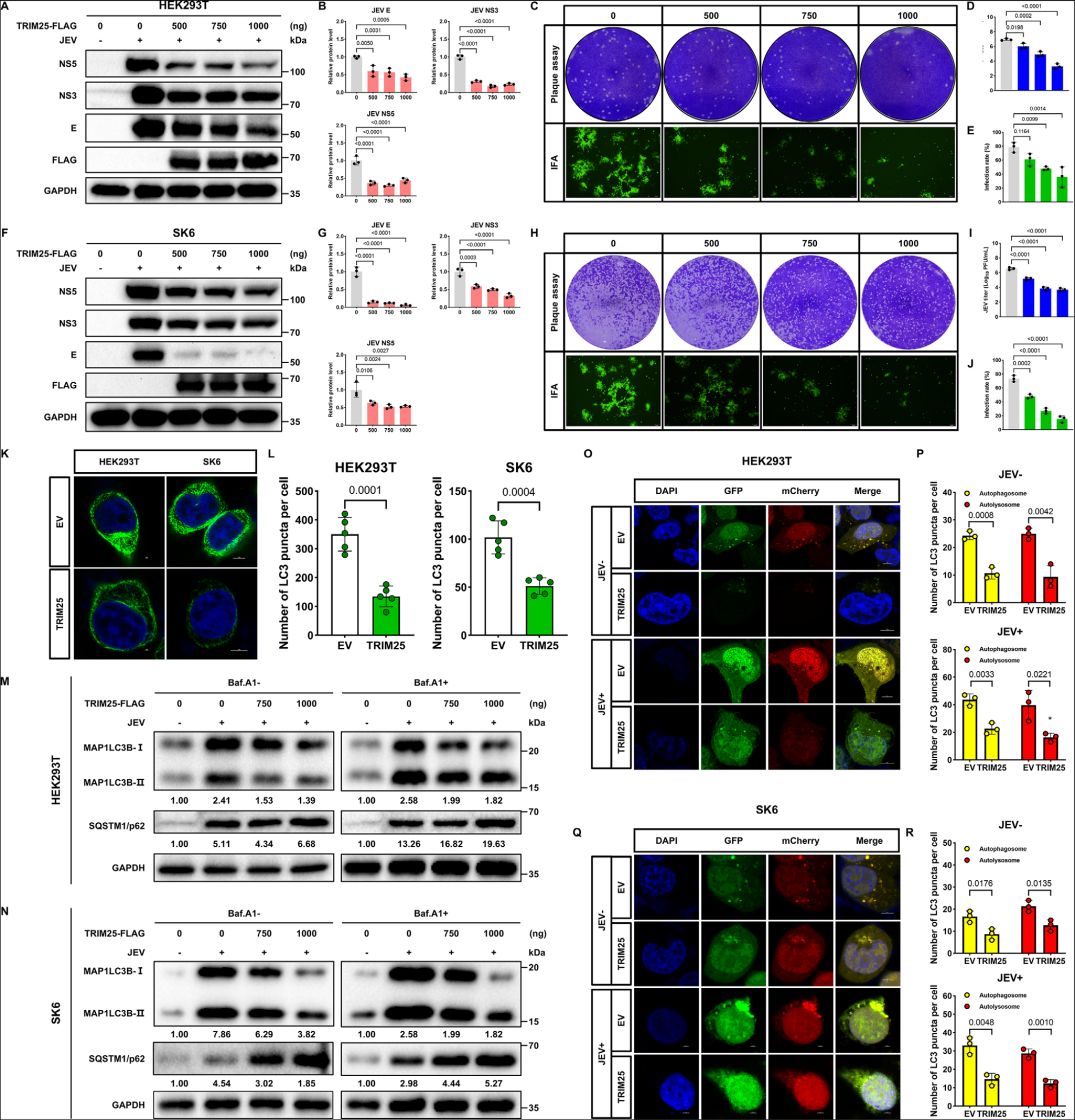
TRIM25 inhibits JEV replication by suppressing autophagic flux. (A) Immunoblot analysis of the expression of JEV E, NS3, and NS5 in HEK293T cells transfected with empty vector control or TRIM25‐FLAG, followed by JEV for 48 h. (B) The expressions of JEV E, NS3, and NS5 were assessed by measuring the band grayscale with the “ImageJ” software. (C) Top: The viral titration analysis of the supernatant in JEV‐infected HEK293T cells expressing TRIM25 was conducted by plaque assay. Bottom: The confocal microscope observation on GFP signal in HEK293T cells transfected with empty vector control or TRIM25‐FLAG, followed by JEV‐GFP infection for 48 h. Scale bar: 100 µm. (D) The statistical analysis of JEV titer in TRIM25‐overexpressed HEK293T cells. (E) The statistical analysis of the percentage of JEV^+^ cells in HEK293T cells transfected with empty vector control or TRIM25‐FLAG. (F) Immunoblot analysis of the expression of JEV E, NS3, and NS5 in SK6 cells transfected with empty vector control or TRIM25‐FLAG, followed by JEV for 48 h. (G) The expressions of JEV E, NS3, and NS5 were assessed by measuring the band grayscale with the “ImageJ” software. (H) Top: The viral titration analysis of the supernatant in JEV‐infected SK6 cells expressing TRIM25 was conducted by plaque assay. Bottom: The confocal microscope observation on GFP signal in SK6 cells transfected with empty vector control or TRIM25‐FLAG, followed by JEV‐GFP infection for 48 h. Scale bar: 100 µm. (I) The statistical analysis of JEV titer in TRIM25‐overexpressed SK6 cells. (J) The statistical analysis of the percentage of JEV^+^ cells in SK6 cells transfected with empty vector control or TRIM25‐FLAG. (K) The confocal microscope observation on GFP‐LC3 puncta formation in HEK293T or SK6 cells transfected with empty vector control or TRIM25‐FLAG, followed by JEV infection for 48 h. Scale bar: 10 µm. (L) The statistical analysis of the number of GFP‐LC3 puncta per cell. (M) Immunoblot analysis of the expression of MAP1LC3B and SQSTM1/p62 in HEK293T cells transfected with empty vector control or TRIM25‐FLAG, followed by JEV for 48 h, with and without Baf.A1 treatment (20 nM). The expressions of MAP1LC3B and SQSTM1/p62 were assessed by measuring the band grayscale with the “ImageJ” software. (N) Immunoblot analysis of the expression of MAP1LC3B and SQSTM1/p62 in SK6 cells transfected with empty vector control or TRIM25‐FLAG, followed by JEV for 48 h, with and without Baf.A1 treatment (20 nM). The expressions of MAP1LC3B and SQSTM1/p62 were assessed by measuring the band grayscale with the “ImageJ” software. (O) The confocal microscope observation on LC3 puncta formation in HEK293T cells transfected with mCherry‐GFP‐LC3 in combination with empty vector control or TRIM25‐FLAG, followed by Mock or JEV infection for 48 h. Red puncta signify autolysosomes, and yellow puncta signify autophagosomes. Scale bar: 5 µm. (P) The statistical analysis of the number of red and yellow LC3 puncta per cell. (Q) The confocal microscope observation on LC3 puncta formation in SK6 cells transfected with mCherry‐GFP‐LC3 in combination with empty vector control or TRIM25‐FLAG, followed by Mock or JEV infection for 48 h. Red puncta signify autolysosomes, and yellow puncta signify autophagosomes. Scale bar: 5 µm. (R) The statistical analysis of the number of red and yellow LC3 puncta per cell. All experiments were conducted in triplicate, and data are represented as mean ± SD. Statistical analysis was performed by a two‐tailed Student's *t*‐test (L, P, and R) or one‐way ANOVA with Tukey's multiple comparisons (B, D, E, G, I, and J).

To further investigate the functional role of TRIM25 in JEV replication, we transfected HEK293T cells with increasing amounts of TRIM25‐FLAG plasmid (0–1000 ng), then infected them with JEV for 48 h. The results revealed that TRIM25 exhibited a potent inhibitory effect on the protein levels of JEV E, NS3, and NS5 (Figure 4A,B). However, silencing TRIM25 enhanced the protein levels of JEV E, NS3, and NS5 (Figure S8A). To further verify this inhibitory effect, viral titers in the supernatants of TRIM25‐overexpressing cells were assessed by plaque assay. Consistent with the immunoblotting data, TRIM25 overexpression resulted in a dose‐dependent decrease in JEV viral loads (Figure 4C, top panel and D). Consistently, TRIM25 overexpression reduced the number of JEV‐positive cells, as assessed by GFP‐tagged virus infection and immunofluorescence analysis (Figure 4C, bottom panel and E). To extend our findings, we also investigated the impact of TRIM25 on JEV replication in SK6 cells. Western blotting, plaque assays, and immunofluorescence analysis consistently demonstrated that TRIM25 potently inhibits JEV replication. (Figure 4F–J).

The E3 ubiquitin ligases of the TRIM family play an emerging role in regulating autophagy under viral infection conditions. To further dissect the mechanistic link between TRIM25 and autophagy in the context of JEV infection, we evaluated the impact of TRIM25 overexpression on key autophagy‐related proteins (ATGs), MAP1LC3B and SQSTM1/p62, in JEV‐infected cells. TRIM25 overexpression significantly diminished cytosolic GFP‐LC3 puncta (Figure 4K,L). Furthermore, Western blot analysis showed that in the presence or absence of the lysosomal inhibitor bafilomycin A1 (Baf.A1), ectopic expression of TRIM25 reduced the levels of the lipidated form of LC3 (LC3ǁ), while increasing the protein level of SQSTM1/p62 compared to controls transfected with an empty vector (Figure 4M,N). Conversely, TRIM25 deficiency led to an increase in LC3ǁ protein and a decrease in SQSTM1/p62 protein levels, which could be rescued by Baf.A1 treatment (Figure S8B,C). During the maturation of autophagic vesicles, autophagosomes fuse with lysosomes to form autolysosomes, which contain active proteases. To monitor the autophagic flux, we employed a tandem mCherry‐GFP‐LC3 reporter construct. In this system, the enhanced green fluorescent protein (eGFP) signal is sensitive to lysosomal proteolysis and is quenched in the acidic environment, whereas the mCherry fluorophore remains stable under acidic conditions. The results indicated that TRIM25 induced a marked reduction in both non‑acidic (mCherry^+^GFP^+^) and acidic (mCherry^+^GFP^−^) puncta in JEV‐infected cells, demonstrating that TRIM25 impairs the formation of both autophagosomes and autolysosomes irrespective of JEV infection (Figure 4O,P). Consistently, we observed identical results, suggesting that TRIM25 disrupts the autophagy process (Figure 4Q,R). These data collectively demonstrated that TRIM25 inhibits JEV replication by suppressing autophagy flux.

### 2.5 TRIM25 Targets ATG7 to Regulate Autophagy Process

**FIGURE 5:**
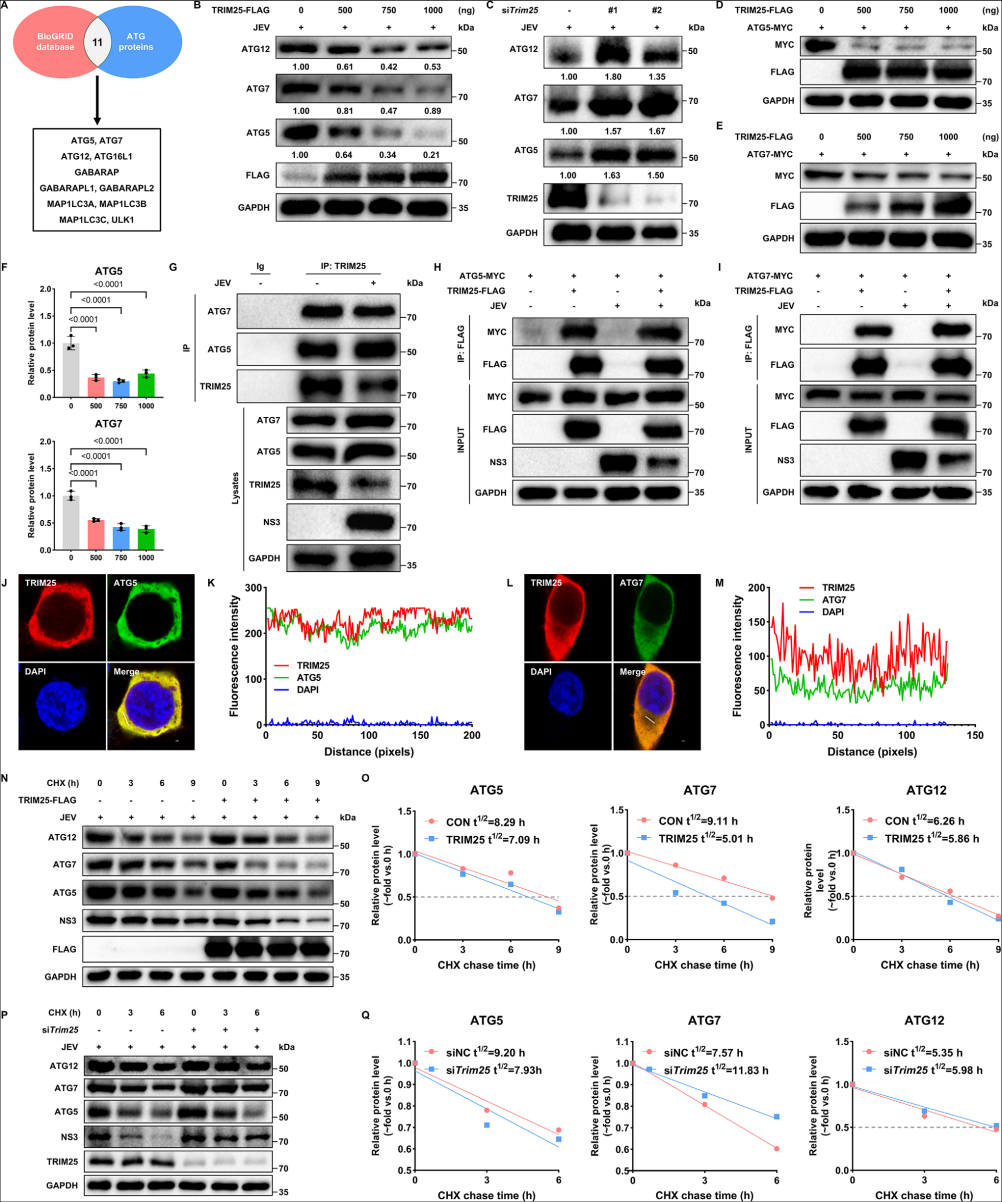
TRIM25 targets ATG7 to regulate autophagy process. (A) The intersection of data derived from the BioGRID database and proteins associated with autophagy pathways, with 11 overlapping proteins being presented. (B) Immunoblot analysis of the expression of ATG5, ATG7, and ATG12 in SK6 cells transfected with empty vector control or TRIM25‐FLAG, followed by JEV for 48 h. The expressions of ATG5, ATG7, and ATG12 were assessed by measuring the band grayscale with the “ImageJ” software. (C) Immunoblot analysis of the expression of ATG5, ATG7, and ATG12 in HEK293T cells transfected with negative control or TRIM25 siRNA, followed by JEV for 48 h. The expressions of ATG5, ATG7, and ATG12 were assessed by measuring the band grayscale with the “ImageJ” software. (D) Immunoblot analysis of the expression of ATG5 in HEK293T cells transfected with TRIM25‐FLAG and ATG5‐MYC, followed by JEV for 48 h. (E) Immunoblot analysis of the expression of ATG7 in HEK293T cells transfected with TRIM25‐FLAG and ATG7‐MYC, followed by JEV for 48 h. (F) The expressions of ATG5 and ATG7 were assessed by measuring the band grayscale with the “ImageJ” software. (G) Immunoblot analysis of the association of TRIM25 with ATG5 and ATG7 by immunoprecipitation of lysates from Mock or JEV‐infected HEK293T cells for 48 h. The cell lysates were immunoprecipitated with anti‐TRIM25 antibodies. (H) Immunoblot analysis of the association of TRIM25 with ATG5 by immunoprecipitation of lysates from HEK293T cells transfected with TRIM25‐FLAG and ATG5‐MYC, followed by Mock or JEV infection for 48 h. The cell lysates were immunoprecipitated with anti‐FLAG antibodies. (I) Immunoblot analysis of the association of TRIM25 with ATG7 by immunoprecipitation of lysates from HEK293T cells transfected with TRIM25‐FLAG and ATG7‐MYC, followed by Mock or JEV infection for 48 h. The cell lysates were immunoprecipitated with anti‐FLAG antibodies. (J) Confocal microscope observation of the colocalization of TRIM25 with ATG5 in HEK293T cells co‐transfected with TRIM25‐FLAG and ATG5‐MYC, followed by JEV infection for 48 h. Scale bar: 1 µm. (K) The colocalization of TRIM25 with ATG5 was quantitatively assessed by measuring the fluorescence intensity with the “ImageJ” software. (L) Confocal microscope observation of the colocalization of TRIM25 with ATG7 in HEK293T cells co‐transfected with TRIM25‐FLAG and ATG7‐MYC, followed by JEV infection for 48 h. Scale bar: 1 µm. (M) The colocalization of TRIM25 with ATG7 was quantitatively assessed by measuring the fluorescence intensity with the “ImageJ” software. (N) Immunoblot analysis of the expressions of ATG5, ATG7, and ATG12 from SK6 cells transfected with TRIM25‐FLAG, followed by cycloheximide (CHX, 50 µg/mL) treatment for the indicated times. (O) The expressions of ATG5, ATG7, and ATG12 were assessed by measuring the band grayscale with the “ImageJ” software. The protein half‐life of ATG5, ATG7, and ATG12 was calculated by nonlinear regression. (P) Immunoblot analysis of the expressions of ATG5, ATG7, and ATG12 from HEK293T cells transfected with negative control or TRIM25 siRNA, followed by CHX (50 µg/mL) treatment for the indicated times. (Q) The expressions of ATG5, ATG7, and ATG12 were assessed by measuring the band grayscale with the “ImageJ” software. The protein half‐life of ATG5, ATG7, and ATG12 was calculated by nonlinear regression. All experiments were conducted in triplicate, and data are represented as mean ± SD. Statistical analysis was performed by one‐way ANOVA with Tukey's multiple comparisons (F).

To identify potential binding ATGs for TRIM25, we first constructed a protein‐protein interaction (PPI) network for TRIM25 using the BioGRID database and intersected the results with proteins involved in autophagy pathways. The findings suggested that ATG5, ATG7, and ATG12 may be regulated by TRIM25 (Figure 5A). To test this, we initially examined the effect of TRIM25 on these ATGs during JEV infection. Western blot analysis revealed that the protein levels of ATG5, ATG7, and ATG12 decreased progressively with higher TRIM25‐FLAG expression (Figure 5B). In contrast, silencing TRIM25 using specific siRNAs increased the protein levels of these ATGs (Figure 5C). To further confirm these observations under exogenous expression conditions, HEK293T cells were co‐transfected with TRIM25‐FLAG and MYC‐tagged ATG5 or ATG7. Consistently, the abundances of ATG5 and ATG7 were reduced in a dose‐dependent manner with increasing TRIM25 expression (Figure 5D–F). Subsequently, we determined whether TRIM25 could interact with ATG proteins to mediate their degradation. The co‐IP analysis showed that TRIM25 could interact with endogenous ATG5 and ATG7 (Figure 5G). In addition, we discovered that TRIM25 could bind with exogenous ATG5 and ATG7 regardless of JEV infection (Figure 5H,I). To further evaluate the interaction between TRIM25 and ATG5/7, confocal microscopy was employed to visualize TRIM25 (displayed in red), ATG5/7 (displayed in green), and nuclei (using DAPI, which appeared blue). The merged images revealed extensive cytoplasmic co‐localization of TRIM25 and ATG5/7. Quantitative analyses of fluorescence intensity and spatial distance further validated their correlative distribution, thereby providing support for a physical interaction between TRIM25 and ATG5/7 (Figure 5J–M). To explore the mechanism by which TRIM25 modulates the expression of ATG proteins, SK6 cells were initially transfected with TRIM25‐FLAG, followed by infection with JEV for 36 h. Then, cells were treated with the protein synthesis inhibitor cycloheximide (CHX) for varying time intervals. Thereafter, the protein degradation rates of multiple ATG proteins were determined. We observed that the degradation of the ATG7 protein was accelerated in TRIM25‐transfected cells. Nevertheless, no significant differences were detected in the degradation of ATG5 or ATG12 proteins between the control (CON) group and the TRIM25‐transfected group (Figure 5N,O). In contrast, silencing TRIM25 prolonged the half‐life of ATG7, not ATG5 or ATG12 (Figure 5P,Q). Taken together, these results indicate that TRIM25 may target ATG7 for degradation to regulate the autophagy process.

### 2.6 TRIM25 Enhances Proteasomal Degradation of ATG7 by Catalyzing K48‐Linked Ubiquitination

**FIGURE 6:**
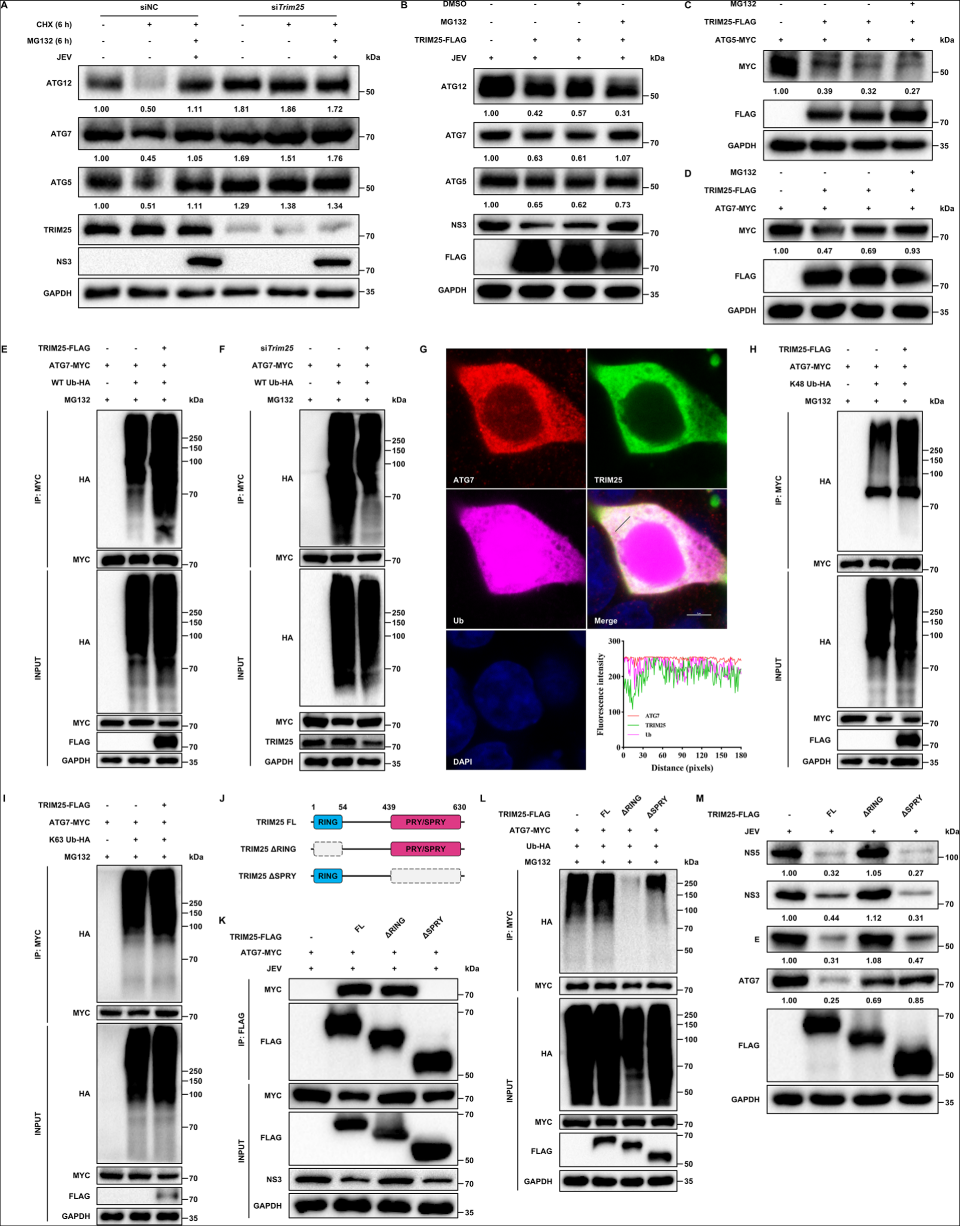
TRIM25 enhances proteasomal degradation of ATG7 by catalyzing K48‐linked ubiquitination. (A) Immunoblot analysis of the expressions of ATG5, ATG7, and ATG12 from HEK293T cells transfected with negative control or TRIM25 siRNA, followed by JEV infection for 36 h. The cells were then treated with CHX (50 µg/mL) and MG132 (10 µM) for 6 h. The expressions of ATG5, ATG7, and ATG12 were assessed by measuring the band grayscale with the “ImageJ” software. (B) Immunoblot analysis of the expressions of ATG5, ATG7, and ATG12 from SK6 cells transfected with empty vector control or TRIM25‐FLAG, followed by JEV infection for 36 h. The cells were then treated with MG132 (10 µM) for 6 h. The expressions of ATG5, ATG7, and ATG12 were assessed by measuring the band grayscale with the “ImageJ” software. (C) Immunoblot analysis of the expression of ATG5 from HEK293T cells transfected with ATG5‐MYC in combination with empty vector control or TRIM25‐FLAG, followed by MG132 (10 µM) treatment for 6 h. The expression of ATG5 was assessed by measuring the band grayscale with the “ImageJ” software. (D) Immunoblot analysis of the expression of ATG7 from HEK293T cells transfected with ATG7‐MYC in combination with empty vector control or TRIM25‐FLAG, followed by MG132 (10 µM) treatment for 6 h. The expression of ATG7 was assessed by measuring the band grayscale with the “ImageJ” software. (E) Immunoblot analysis of the effect of TRIM25 on ATG7 ubiquitination from HEK293T cells co‐transfected with TRIM25‐FLAG, ATG7‐MYC, and Ub‐HA, followed by MG132 (10 µM) treatment for 6 h. The lysates were subjected to precipitation using anti‐MYC antibodies for the enrichment of ubiquitinated proteins, followed by probing with the specified antibodies. (F) Immunoblot analysis of the effect of TRIM25 on ATG7 ubiquitination from HEK293T cells co‐transfected with TRIM25 siRNA, ATG7‐MYC, and Ub‐HA, followed by MG132 (10 µM) treatment for 6 h. The lysates were subjected to precipitation using anti‐MYC antibodies for the enrichment of ubiquitinated proteins, followed by probing with the specified antibodies. (G) Confocal microscope observation of the colocalization of TRIM25, ATG7, and Ubi in HEK293T cells co‐transfected with TRIM25‐EGFP, ATG7‐MYC, and Ub‐HA, followed by MG132 (10 µM) treatment for 6 h. Scale bar: 5 µm. (H) Immunoblot analysis of the effect of TRIM25 on ATG7 ubiquitination from HEK293T cells co‐transfected with TRIM25 siRNA, ATG7‐MYC, and K48 Ub‐HA, followed by MG132 (10 µM) treatment for 6 h. The lysates were subjected to precipitation using anti‐MYC antibodies for the enrichment of ubiquitinated proteins, followed by probing with the specified antibodies. (I) Immunoblot analysis of the effect of TRIM25 on ATG7 ubiquitination from HEK293T cells co‐transfected with TRIM25 siRNA, ATG7‐MYC, and K63 Ub‐HA, followed by MG132 (10 µM) treatment for 6 h. The lysates were subjected to precipitation using anti‐MYC antibodies for the enrichment of ubiquitinated proteins, followed by probing with the specified antibodies. (J) Schematic graph of the structural regions of TRIM25 (full length, FL) and its truncations (ΔRING and ΔSPRY). (K) Immunoblot analysis of the association of TRIM25 structural regions with ATG7 by immunoprecipitation of lysates from HEK293T cells transfected with ATG7‐MYC in combination with TRIM25‐FLAG FL, ΔRING, and ΔSPRY, followed by JEV infection for 48 h. The cell lysates were immunoprecipitated with anti‐FLAG antibodies. (L) Immunoblot analysis of the effect of TRIM25 structural regions on ATG7 ubiquitination from HEK293T cells co‐transfected with ATG7‐MYC and Ub‐HA in combination with TRIM25‐FLAG FL, ΔRING, and ΔSPRY, followed by MG132 (10 µM) treatment for 6 h. The lysates were subjected to precipitation using anti‐MYC antibodies for the enrichment of ubiquitinated proteins, followed by probing with the specified antibodies. (M) Immunoblot analysis of the expressions of JEV NS3, NS5, and ATG7 from SK6 cells transfected with empty vector control or TRIM25‐FLAG FL, ΔRING, and ΔSPRY mutant plasmids, followed by JEV infection for 48 h. The expressions of JEV NS3, NS5, and ATG7 were assessed by measuring the band grayscale with the “ImageJ” software. All experiments were conducted in triplicate, and data are represented as mean ± SD.

Given that our study indicated that TRIM25 subverts autophagy activation by suppressing ATG proteins, we aim to elucidate the mechanism by which TRIM25 drives the degradation of ATG proteins. Owing to TRIM25 being an E3 ubiquitin ligase known to regulate protein stability via ubiquitination, we hypothesized that TRIM25 might subvert the autophagy process by mediating ATG protein degradation through the ubiquitin‐proteasome system (UPS). HEK293T cells were transfected with either NC or TRIM25 siRNA, and subsequently treated with CHX at a concentration of 50 µg/mL or MG132 at 10 µM for 0 or 6 h. The results showed a significant decrease in the abundances of ATG5, ATG7, and ATG12 proteins following CHX treatment. Additionally, MG132 was able to reverse the degradation of endogenous ATG5, ATG7, and ATG12. However, the knockdown of TRIM25 negated this effect (Figure 6A). To confirm that TRIM25‐mediated ubiquitination of ATG proteins leads to their degradation, we ectopically expressed the TRIM25‐FLAG plasmid in SK6 cells, followed by infection with JEV for 36 h. The cells were then treated with MG132 (10 µM) for 6 h. The results indicated that TRIM25 overexpression led to a significant reduction in the protein level of ATG7. Moreover, MG132 treatment rescued the reduction of endogenous ATG7, rather than ATG5 or ATG12 proteins, induced by TRIM25 (Figure 6B). Further, we found that the impairment of exogenous ATG7, not ATG5, induced by TRIM25 was abolished by MG132 treatment (Figure 6C,D).

Next, ubiquitination assays were conducted to ascertain whether TRIM25 facilitates the ubiquitination of ATG7. HEK293T cells were transfected with TRIM25‐FLAG plasmids, followed by MG132 treatment. The co‐IP analysis revealed that the ubiquitination of endogenous ATG7 was augmented with the expression of TRIM25, yet diminished upon the silencing of TRIM25 (Figure S9A,B). Additionally, we observed that the ubiquitination of exogenous ATG7 also increased with TRIM25 expression but decreased with TRIM25 silencing (Figure 6E,F). Confocal analysis demonstrated a significant colocalization of TRIM25, ATG7, and Ubiquitin in the cytoplasm (Figure 6G). Subsequently, we delved into the specific subtypes of ubiquitin chains implicated in the ubiquitination of ATG7 mediated by TRIM25. Among the various ubiquitination mechanisms, K48‐linked and K63‐linked ubiquitination stand out as the most prevalent degradation‐associated pathways within the realm of ubiquitin‐mediated processes. Our findings revealed that the K48 mutation, as opposed to the K63 mutation, interacted efficiently with ATG7, suggesting the direct catalytic impact of TRIM25 on K48‐linked ATG7 ubiquitination (Figure 6H,I, and Figure S9C,D).

Since TRIM25 is part of the TRIM family, the majority of its members are RING‐type E3 ligases; we focus on its E3 ligase activity, which might play a negative regulatory role on ATG7 protein levels. Initially, we created domain‐deficient mutants (ΔRING and ΔSPRY) (Figure 6J) and co‐transfected HEK293T cells with FLAG‐TRIM25 WT, either the ΔRING or ΔSPRY mutant plasmid, along with a MYC‐ATG7 plasmid, followed by JEV infection. Our domain‐specific truncation analysis of TRIM25 revealed that ATG7 binds to its SPRY domain, rather than the RING domain (Figure 6K). Next, we conducted ubiquitination assays, which demonstrated that the expression of FLAG‐TRIM25 FL but not that of FLAG‐TRIM25 ΔRING mutant plasmid significantly increased the ubiquitination of ATG7 in HEK293T cells (Figure 6L). Consistent with this observation, the protein level of ATG7 was significantly reduced in cells overexpressing TRIM25, but not in those transfected with FLAG‐TRIM25 ΔRING or ΔSPRY mutant plasmid. Intriguingly, only the deletion of the RING domain allowed JEV replication to proceed (Figure 6M). Therefore, these findings demonstrated that TRIM25 facilitates the ubiquitination of ATG7 in a way reliant on its RING domain.

### 2.7 TRIM25 Promotes Ubiquitination of ATG7 at Lysine 389 and 423

**FIGURE 7:**
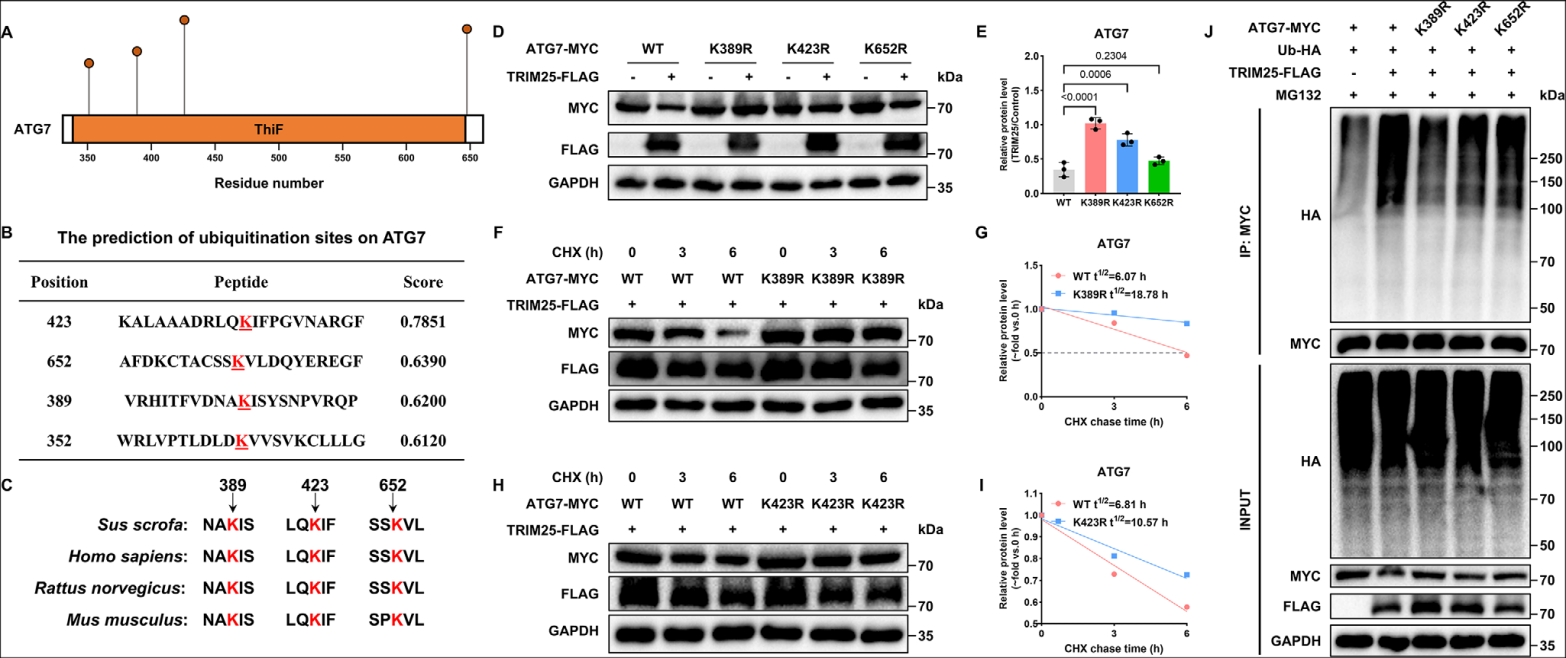
TRIM25 promotes ubiquitination of ATG7 at lysine 389 and 423. (A) Schematic graph of the ubiquitination sites of ATG7. (B) In silico TRIM25‐specific prediction of ubiquitination sites on ATG7 on the GPS‐Uber website (https://gpsuber.biocuckoo.cn/index.php). (C) The lysine residues at 389, 423, and 652 of ATG7 are evolutionarily conserved across swine (*Sus scrofa*), Human (*Homo sapiens*), rat (*Rattus norvegicus*), and mouse (*Mus musculus*). (D) Immunoblot analysis of the expression of ATG7 from HEK293T cells transfected with ATG7‐MYC WT, K389R, K423R, and K652R in combination with empty vector control or TRIM25‐FLAG. (E) The expression of ATG7 was assessed by measuring the band grayscale with the “ImageJ” software. (F) Immunoblot analysis of the expression of ATG7 from HEK293T cells transfected with TRIM25‐FLAG in combination with ATG7‐MYC WT or K389R, followed by CHX (50 µg/mL) treatment for the indicated times. (G) The expression of ATG7 was assessed by measuring the band grayscale with the “ImageJ” software. The protein half‐life of ATG7 WT or K389R was calculated by nonlinear regression. (H) Immunoblot analysis of the expression of ATG7 from HEK293T cells transfected with TRIM25‐FLAG in combination with ATG7‐MYC WT or K423R, followed by CHX (50 µg/mL) treatment for the indicated times. (I) The expression of ATG7 was assessed by measuring the band grayscale with the “ImageJ” software. The protein half‐life of ATG7 WT or K423R was calculated by nonlinear regression. (J) Immunoblot analysis of the effect of TRIM25 on ATG7 WT, K389R, K423R, and K652R ubiquitination from HEK293T cells co‐transfected with ATG7‐MYC WT, K389R, K423R, and K652R and Ubi‐HA in combination with TRIM25‐FLAG, followed by MG132 (10 µM) treatment for 6 h. The lysates were subjected to precipitation using anti‐MYC antibodies for the enrichment of ubiquitinated proteins, followed by probing with the specified antibodies. All experiments were conducted in triplicate, and data are represented as mean ± SD. Statistical analysis was performed by one‐way ANOVA with Tukey's multiple comparisons (E).

To map the ubiquitination sites on ATG7, we make a TRIM25‐specific prediction on the GPS‐Uber website (https://gpsuber.biocuckoo.cn/index.php). According to the prediction score, we selected Lys423, Lys652, and Lys389 for the next study (Figure 7A,B). Moreover, the Lys389, 423, and 652 sites are evolutionarily conserved in the ATG7 protein among pig, human, mouse, and rat (Figure 7C). Then, we generated a series of ATG7 mutants by substituting lysine (K) residues with arginine (R) at positions 389, 423, and 652 (ATG7‐K389R, ATG7‐K423R, ATG7‐K652R) to investigate the effect of TRIM25 on ATG7 protein. Our results indicated that TRIM25 overexpression significantly reduced the protein levels of ATG7, while the K389R and K423R mutants exhibited resistance to degradation induced by TRIM25 (Figure 7D,E). Additionally, after transfection with the MYC‐ATG7 (WT) plasmid and MYC‐ATG7 (K389R or K423R) plasmid individually, HEK293T cells were treated with CHX for 0, 3, and 6 h. Western blotting results indicated that TRIM25‐induced ATG7 degradation was halted in cells with the K389R and K423R mutation in ATG7 (Figure 7F–I). Next, we examined the polyubiquitination associated with these mutants in HEK293T cells. Lysine mutation at 389 and 423 sites abrogated the enhancement of ATG7 ubiquitination induced by TRIM25 (Figure 7J). These findings imply that the K389 and K423 sites of ATG7 are responsible for TRIM25‐triggered ubiquitination and degradation.

### 2.8 ZNF33B Promotes JEV Replication by Alleviating TRIM25‐Mediated Suppression of Autophagy

**FIGURE 8:**
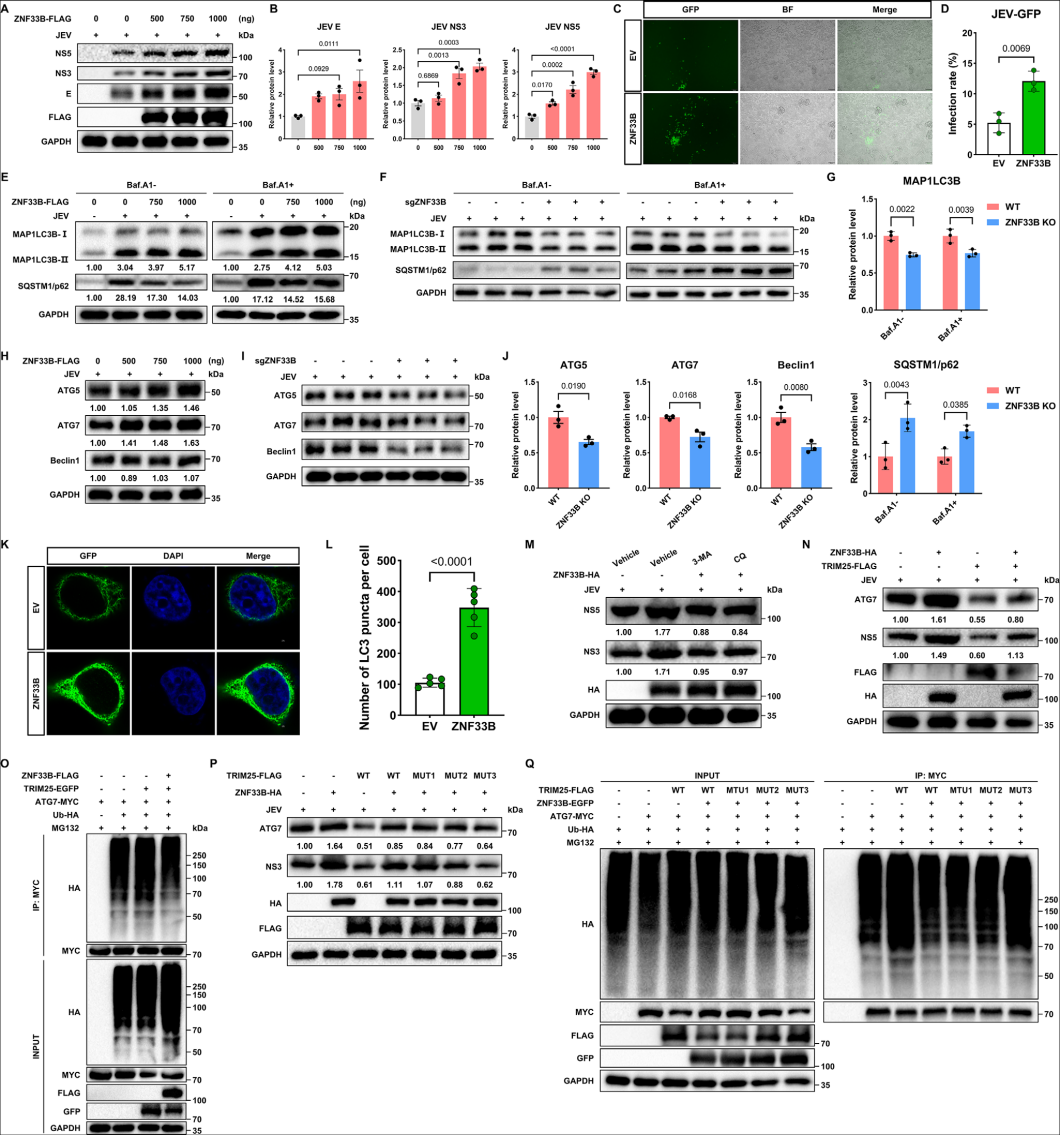
ZNF33B promotes JEV replication by alleviating TRIM25‐mediated suppression of autophagy. (A) Immunoblot analysis of the expression of JEV E, NS3, and NS5 in SK6 cells transfected with empty vector control or ZNF33B‐FLAG, followed by JEV for 48 h. (B) The expressions of JEV E, NS3, and NS5 were assessed by measuring the band grayscale with the “ImageJ” software. (C) The confocal microscope observation on GFP signal in SK6 cells transfected with empty vector control or ZNF33B‐FLAG, followed by JEV‐GFP infection for 48 h. Scale bar: 100 µm. (D) The statistical analysis of the percentage of JEV^+^ cells in SK6 cells transfected with empty vector control or ZNF33B‐FLAG. (E) Immunoblot analysis of the expression of MAP1LC3B and SQSTM1/p62 in HEK293T cells transfected with empty vector control or ZNF33B‐FLAG, followed by JEV for 48 h, with and without Baf.A1 treatment (20 nM). The expressions of MAP1LC3B and SQSTM1/p62 were assessed by measuring the band grayscale with the “ImageJ” software. (F) Immunoblot analysis of the expression of MAP1LC3B and SQSTM1/p62 in PK15 WT and ZNF33B KO cells infected by JEV for 48 h, with and without Baf.A1 treatment (20 nM). (G) The expressions of MAP1LC3B and SQSTM1/p62 were assessed by measuring the band grayscale with the “ImageJ” software. (H) Immunoblot analysis of the expression of ATG5, ATG7, and Beclin1 in SK6 cells transfected with empty vector control or ZNF33B‐FLAG, followed by JEV for 48 h. The expressions of ATG5, ATG7, and Beclin1 were assessed by measuring the band grayscale with the “ImageJ” software. (I) Immunoblot analysis of the expression of ATG5, ATG7, and Beclin1 in JEV‐infected PK‐15 WT and *Znf33b* ^−/−^ cells for 48 h. (J) The expressions of ATG5, ATG7, and Beclin1 were assessed by measuring the band grayscale with the “ImageJ” software. (K) The confocal microscope observation on GFP‐LC3 puncta formation in SK6 cells transfected with empty vector control or ZNF33B‐FLAG, followed by JEV infection for 48 h. Scale bar: 1 µm. (L) The statistical analysis of the number of GFP‐LC3 puncta per cell. (M) Immunoblot analysis of the expressions of JEV NS3 and NS5 in SK6 cells transfected with empty vector control or ZNF33B‐FLAG, followed by JEV infection for 36 h. The cells were then treated with 3‐MA (10 mM) and CQ (10 µM) for 12 h. The expressions of JEV NS3 and NS5 were assessed by measuring the band grayscale with the “ImageJ” software. (N) Immunoblot analysis of the expression of ATG7 and JEV NS5 in HEK293T cells transfected with ZNF33B‐HA and TRIM25‐FLAG, followed by JEV for 48 h. The expressions of ATG7 and JEV NS5 were assessed by measuring the band grayscale with the “ImageJ” software. (O) Immunoblot analysis of the effect of ZNF33B on TRIM25‐promoted ATG7 ubiquitination from HEK293T cells co‐transfected with ATG7‐MYC, Ub‐HA ZNF33B‐FLAG, and TRIM25‐EGFP, followed by MG132 (10 µM) treatment for 6 h. The lysates were subjected to precipitation using anti‐MYC antibodies for the enrichment of ubiquitinated proteins, followed by probing with the specified antibodies. (P) Immunoblot analysis of the expression of ATG7 and JEV NS3 in SK6 cells transfected with ZNF33B‐HA and TRIM25‐FLAG WT, MUT1, MUT2, and MUT3, followed by JEV for 48 h. The expressions of ATG7 and JEV NS3 were assessed by measuring the band grayscale with the “ImageJ” software. (Q) Immunoblot analysis of the effect of ZNF33B on TRIM25‐promoted ATG7 ubiquitination from HEK293T cells co‐transfected with ATG7‐MYC, Ub‐HA ZNF33B‐EGFP in combination with TRIM25‐FLAG WT, MUT1, MUT2, and MUT3, followed by MG132 (10 µM) treatment for 6 h. The lysates were subjected to precipitation using anti‐MYC antibodies for the enrichment of ubiquitinated proteins, followed by probing with the specified antibodies. All experiments were conducted in triplicate, and data are represented as mean ± SD. Statistical analysis was performed by a two‐tailed Student's *t*‐test (D, G, J, and L) or one‐way ANOVA with Tukey's multiple comparisons (B).

To investigate the effect of ZNF33B on JEV replication, SK6 cells were transfected with ZNF33B‐FLAG, then subjected to JEV infection for 48 h. The results revealed that the abundances of E, NS3, and NS5 proteins remarkably increased under ZNF33B overexpression (Figure 8A,B). Next, we created a JEV‐GFP by fusing the virus with green fluorescent protein and infected the ZNF33B‐overexpressing cells. We observed a significant increase in the JEV‐positive cells upon overexpression of ZNF33B (Figure 8C,D). In addition to JEV, we also detected the effect of ZNF33B on the replication of other *Flaviviridae* viruses. Interestingly, overexpressing ZNF33B increased the protein level of ZIKV NS3. Depleting ZNF33B, on the other hand, inhibited its replication (Figure S10A,C). Moreover, the IFA assay showed that overexpressing ZNF33B enhanced the fluorescence intensity of ZIKV NS3 in SK6 cells (Figure S10B). On the contrary, the fluorescence intensity of ZIKV NS3 was reduced by depleting ZNF33B (Figure S10D). However, the level of CSFV E2 was comparable whether ZNF33B was overexpressed or depleted (Figure S10E–H). Collectively, these findings implied that ZNF33B exerts a beneficial influence on Flavivirus replication.

Our previous research suggested that the autophagy agonist rapamycin enhances JEV replication. We then determined the effect of ZNF33B on autophagy in JEV‐infected cells. The results demonstrated that the protein levels of LC3ǁ increased while SQSTM1/p62 decreased by ZNF33B overexpression (Figure 8E). Moreover, the depletion of ZNF33B significantly inhibited the protein levels of LC3ǁ and enhanced the protein levels of SQSTM1/p62 in JEV‐infected cells (Figure 8F,G). Moreover, the protein levels of ATG5, ATG7, and Beclin1 increased by ZNF33B overexpression (Figure 8H). Depletion of ZNF33B significantly inhibited the protein levels of ATG5, ATG7, and Beclin1 in JEV‐infected cells (Figure 8I,J). Next, the data revealed a consistent and significant increase in cytosolic GFP‐LC3 puncta upon overexpression of ZNF33B (Figure 8K,L). In summary, our results implied that ZNF33B is critical for autophagy induction in JEV‐infected cells. To confirm the role of autophagy in ZNF33B‐mediated JEV replication, SK6 cells were transfected with ZNF33B‐HA and treated with the autophagy inhibitor 3‐methyladenine (3‐MA) or chloroquine (CQ) before JEV infection. Western blot results showed that 3‐MA or CQ treatment significantly reduced the ZNF33B‐induced upregulation of JEV NS3 and NS5 proteins (Figure 8M). To further dissect the mechanistic interplay between ZNF33B, TRIM25, and autophagy in mediating JEV replication, rescue experiments were performed. First, ZNF33B‐HA and TRIM25‐FLAG plasmids were co‐expressed in HEK293T cells, then infected with JEV for 48 h. Western blot analysis revealed that overexpression of ZNF33B remarkably reversed the TRIM25‐induced downregulation of JEV NS3 and ATG7 proteins (Figure 8N). Given that TRIM25 inhibits autophagy by targeting ATG7 for UPS‐mediated degradation, we next investigated whether ZNF33B modulates ATG7 ubiquitination through TRIM25. The results showed that TRIM25 overexpression increased the ubiquitination of ATG7, while co‐expression of ZNF33B abrogated these effects (Figure 8O).

To determine whether ZNF33B benefits JEV replication by regulating the m^6^A‐TRIM25‐autophagy axis, ZNF33B‐HA in combination with TRIM25‐FLAG (including wild type, m^6^A sites mutations MUT1, MUT2, and MUT3) plasmids were delivered into SK6 cells, followed by JEV infection for 48 h. The results revealed that ZNF33B enhances the protein abundances of JEV NS3 and ATG7 by antagonizing TRIM25. However, this ability was lost in the cells transfected with TRIM25 MUT3 (Figure 8P), supporting the results that ZNF33B targets m^6^A‐modified sites in the *Trim25* mRNA to promote its degradation (Figure 3G–I). We then assessed the ubiquitination levels of ATG7. Our findings revealed that overexpression of ZNF33B impeded TRIM25‐induced the enhancement of ATG7 ubiquitination. Interestingly, TRIM25 MUT3 still retained the ability to enhance the ubiquitination level of ATG7, indicating that ZNF33B disrupts TRIM25‐mediated ATG7 ubiquitination through m^6^A modification (Figure 8Q). Taken together, these results reveal that ZNF33B promotes JEV replication by alleviating TRIM25‐mediated suppression of autophagy.

### 2.9 ZNF33B Exacerbates JEV Neuropathogenesis In Vivo

**FIGURE 9:**
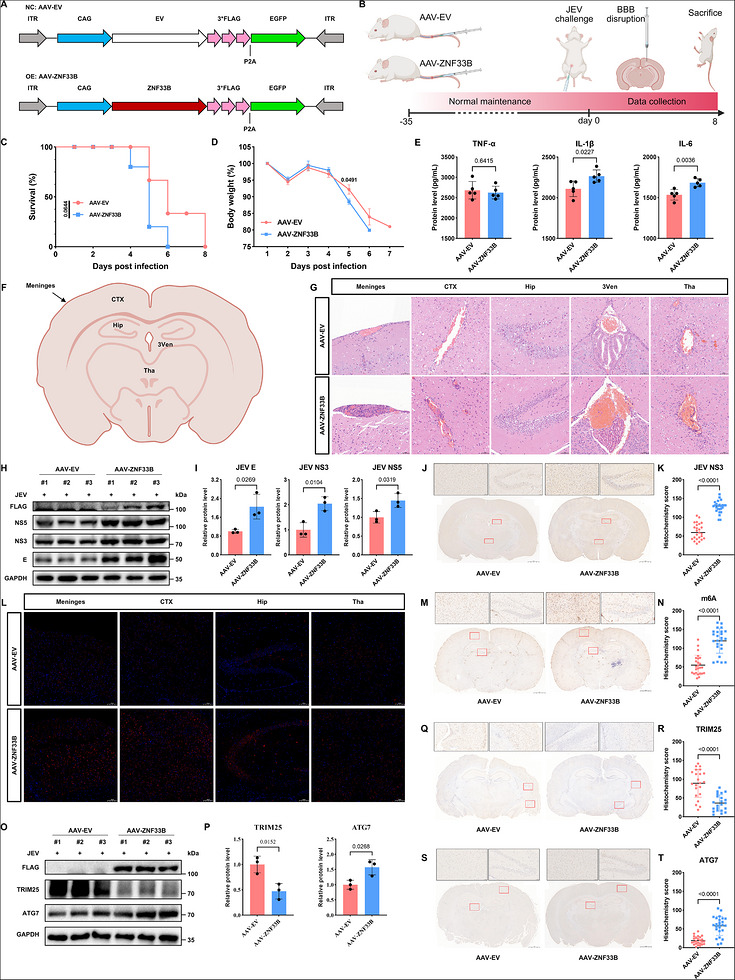
ZNF33B exacerbates JEV neuropathogenesis in vivo. (A) Schematic diagram of AAV‐EV and AAV‐ZNF33B structures. (B) 7‐week‐old female mice were intravenously (i.v.) injected with AAV‐EV (n = 5) or AAV‐ZNF33B (n = 5). After 35 days (when the mice were 12 weeks old), the mice were infected with the JEV P3 strain by intraperitoneal injection. The blood‐brain barrier (BBB) was then disrupted immediately using a microsyringe. The diagram was generated using Biorender.com. (C) Kaplan‐Meier survival of brain‐specific ZNF33B‐overexpressing mice infected with JEV P3 strain. (D) Percentage of weight loss of brain‐specific ZNF33B‐overexpressing mice infected with JEV P3 strain. (e) The levels of TNF‐α, IL‐1β, and IL‐6 in brain‐specific ZNF33B‐overexpressing mice infected with JEV P3 strain were detected by ELISA. (F) Schematic representation of different sections of the mouse brain. (G) Hematoxylin and eosin staining of brain tissue sections from brain‐specific ZNF33B‐overexpressing mice infected with JEV P3 strain. Scale bar: 50 µm. (H) Immunoblot analysis of the expression of JEV E, NS3, and NS5 in brain tissue homogenate lysates from brain‐specific ZNF33B‐overexpressing mice infected with JEV P3 strain. (I) The expressions of JEV E, NS3, and NS5 were assessed by measuring the band grayscale with the “ImageJ” software. (J) Immunohistochemical (IHC) staining of JEV NS3 in brain tissue sections from brain‐specific ZNF33B‐overexpressing mice infected with JEV P3 strain. Scale bar: 100 µm. (K) The level of JEV NS3 was assessed by measuring the immunohistochemical score from 25 different areas with the “Saiviewer” software. (L) Immunofluorescence analysis (IFA) of JEV NS3 in brain tissue sections from brain‐specific ZNF33B‐overexpressing mice infected with JEV P3 strain. Scale bar: 100 µm. (M) IHC staining of m6A in brain tissue sections from brain‐specific ZNF33B‐overexpressing mice infected with JEV P3 strain. Scale bar: 100 µm. (N) The level of m6A was assessed by measuring the immunohistochemical score from 25 different areas with the “Saiviewer” software. (O) Immunoblot analysis of the expression of TRIM25 and ATG7 in brain tissue homogenate lysates from brain‐specific ZNF33B‐overexpressing mice infected with JEV P3 strain. (P) The expressions of JEV TRIM25 and ATG7 were assessed by measuring the band grayscale with the “ImageJ” software. (Q) IHC staining of TRIM25 in brain tissue sections from brain‐specific ZNF33B‐overexpressing mice infected with JEV P3 strain. Scale bar: 100 µm. (R) The level of TRIM25 was assessed by measuring the immunohistochemical score from 25 different areas with the “Saiviewer” software. (S) IHC staining of ATG7 in brain tissue sections from brain‐specific ZNF33B‐overexpressing mice infected with JEV P3 strain. Scale bar: 100 µm. (T) The level of ATG7 was assessed by measuring the immunohistochemical score from 25 different areas with the “Saiviewer” software. All experiments were conducted in triplicate, and data are represented as mean ± SD. Statistical analysis was performed by a two‐tailed Student's *t*‐test (C, D, E, I, K, N, P, R, and T).

To validate the in vitro findings in an in vivo model, we established a JEV‐infected mouse model. Balb/c mice were intravenously (i.v.) injected via tail vein with adeno‐associated virus expressing ZNF33B (AAV‐ZNF33B, n = 5 mice) or control adeno‐associated virus (AAV‐EV, n = 5 mice) to mimic ZNF33B transgenic models. 35 days later, the mice were intraperitoneally (i.p.) infected with JEV P3 strain, and the survival rate, clinical symptoms, and viral level in the brain were monitored (Figure 9A,B). During the 8‐day observation period after JEV infection, AAV‐ZNF33B‐infected mice exhibited more severe clinical symptoms (such as hindlimb paralysis, tremors) compared to AAV‐EV‐infected mice. Importantly, AAV‐ZNF33B treatment significantly advanced the mortality time and increased weight loss in JEV‐infected mice, as indicated by the survival curve and percentage of body weight (Figure 9C,D). Moreover, AAV‐ZNF33B resulted in hyperinflammation by raising IL‐1β and IL‐6 levels during JEV infection (Figure 9E). The hematoxylin‐eosin (HE) staining indicated that mice in the AAV‐ZNF33B group showed more severe pathological damage in the brain, including brain tissue edema, massive degeneration and necrosis of nerve cells, a significant decrease in the number of neurons, and a large number of inflammatory cell infiltrations, which were mainly concentrated in the cerebral cortex and hippocampus (Figure 9F,G).

Subsequently, we determined the relative content of JEV in the brain by WB, IFA, and immunohistochemical (IHC) assays. The findings indicated that AAV‐ZNF33B brought about a remarkable increase in the abundances of both JEV E, NS3, and NS5 proteins (Figure 9H,I). In addition, both IHC and IFA results indicated a significant JEV NS3 positive signal in the AAV‐ZNF33B group, indicating that ZNF33B could significantly promote the replication of JEV in the mouse brain (Figure 9J–L). The global m^6^A modification level in mouse brain tissues was also examined. The results showed that the global m^6^A modification level in the brain tissues of mice in the AAV‐ZNF33B group was significantly higher than that in the AAV‐EV group, indicating that ZNF33B could increase the global m^6^A modification level in the brain tissue in vivo (Figures 9M,N, and S11). Furthermore, we detected TRIM25 and ATG7 protein levels in the brain tissues of JEV‐infected mice. Western blot analysis showed that overexpression of ZNF33B decreased the TRIM25 protein level and increased the ATG7 protein level in the brain (Figure 9O,P). Further, the IHC assay was used to detect the expression and localization of TRIM25 and ATG7 in mouse brain tissues. The results showed that TRIM25 was mainly expressed in the cytoplasm of neurons, and the number of TRIM25‐positive cells in the brain tissues of mice in the AAV‐ZNF33B group was significantly less than that in the AAV‐EV group (Figure 9Q,R); ATG7 was also mainly expressed in the cytoplasm of neurons, and the number of ATG7‐positive cells in the brain tissues of mice in the AAV‐ZNF33B group was significantly more than that in the AAV‐EV group (Figure 9S,T). Collectively, these results indicated that in vivo, ZNF33B promotes JEV replication by regulating the m^6^A‐TRIM25‐ATG7 axis.

## 3 Discussion

As an important zoonotic viral encephalitis pathogen, JEV poses a serious threat to human health and the development of animal husbandry [30, 31]. However, the host pathways that are manipulated to support its replication remain incompletely understood. In this study, we identify a previously unrecognized mechanism by which JEV exploits host epitranscriptomic regulation to reprogram autophagy and evade antiviral defenses. We demonstrate that the C_2_H_2_ zinc‐finger protein ZNF33B functions as a proviral host factor by integrating m^6^A RNA modification with ubiquitin‐mediated control of autophagy through selective destabilization of *Trim25* mRNA. These findings establish a ZNF33B‐m^6^A‐TRIM25‐ATG7 axis that links RNA methylation, innate immunity, and autophagic flux in flaviviral infection.

The m^6^A modification has emerged as a critical node in virus‐host interplay, with diverse viruses hijacking the host methylation machinery for optimal replication [32, 33]. Notably, several RNA viruses, including HIV, influenza virus, and Zika virus, have been shown to exploit or alter the host m^6^A machinery to favor their replication [10, 21, 22]. Consistent with this paradigm, we observed that JEV infection enhances the abundance of the m^6^A writer components METTL3 and METTL14. Importantly, our data identify ZNF33B as a previously uncharacterized regulator of m^6^A deposition. Rather than altering METTL3 or METTL14 expression, ZNF33B physically associates with METTL14 and promotes stabilization of the METTL3‐METTL14 heterodimer, thereby amplifying global m^6^A levels. This mechanism expands current understanding of how RNA‐binding proteins fine‐tune m^6^A activity, complementing prior studies describing ZC3H13, which has been reported to bridge the m^6^A writer complex to nuclear RNA, while other ZNF family members participate in splicing and stability control [34, 35, 36]. Our findings suggest that ZNF33B acts as an epitranscriptomic scaffold that selectively potentiates m^6^A‐dependent post‐transcriptional regulation during viral infection, extending our understanding of how viruses exploit host epitranscriptomic machinery beyond known immune‐regulated mechanisms.

Through multiomic analyses (RIP‐seq and RNA‐seq), we identified that ZNF33B exhibited stronger binding affinity for *Trim25* compared to other identified targets (*Dhx58*, *Znfx1*). TRIM25 is a well‐established antiviral E3 ubiquitin ligase that activates RIG‐I‐mediated interferon signaling by catalyzing K63‐linked ubiquitination of RIG‐I [37]. Conversely, multiple viruses, including the Influenza A virus NS1 protein and SARS‐CoV‐2 nucleocapsid protein, antagonize its activity to dampen innate immunity [38, 39]. Here, our study uncovers a distinct mechanism of TRIM25 function, demonstrating that ZNF33B selectively recognizes m^6^A‐modified sites within *Trim25* mRNA and accelerates its decay, thereby reducing TRIM25 protein abundance. The specificity of this interaction is underscored by the requirement for two conserved m^6^A sites (c.1567 and c.1669), whose mutation disrupts ZNF33B binding and restores TRIM25 stability. This is supported by the findings that METTL3/METTL14 overexpression suppressed TRIM25 protein levels, while DAA treatment or FTO overexpression reversed ZNF33B‐mediated TRIM25 downregulation. This mechanism reveals how viruses can exploit host epitranscriptomic machinery not merely to enhance viral RNA metabolism, but to selectively dismantle antiviral gene expression programs. Given that ZNF33B binds the “RGAC” m^6^A consensus motif, its target repertoire likely extends beyond *Trim25* to include other defense transcripts, as suggested by our RIP‐seq data implicating *Dhx58* and *Znfx1*. The global m^6^A hypermodification induced by ZNF33B could therefore represent a systemic rewiring of host transcriptome stability that favors viral propagation.

Beyond its classical role in interferon signaling, we found that TRIM25 acts as a potent inhibitor of autophagy, a process increasingly recognized for its complex, context‐dependent roles in viral infections. Emerging evidence indicates that TRIM family proteins are versatile regulators of autophagy. TRIM28 acts as a molecular scaffold to enhance Vps34‐Beclin1 interaction and promote autophagy [40]. While autophagy can degrade viral components (xenophagy), many flaviviruses, including JEV, DENV, and HCV, co‐opt autophagic membranes to facilitate their replication [21, 22, 23]. Our results demonstrate that TRIM25 suppresses autophagy by targeting ATG7 for ubiquitin‐mediated proteasomal degradation, emphasizing the pivotal role of ubiquitination in regulating autophagic flux. Specifically, TRIM25 catalyzes K48‐linked ubiquitination of ATG7 at lysine residues 389 and 423, leading to reduced ATG7 stability and impaired autophagic flux. However, we do not exclude the possibility that the canonical innate immunity pathway also contributes to TRIM25‐mediated restriction of JEV. Rather, our findings reveal a parallel mechanism‐K48‐linked ubiquitination and degradation of ATG7‐that operates in concert with IFN signaling to suppress JEV replication. The relative contribution of these two pathways may depend on the cellular context, viral load, and stage of infection. Our finding expands the functional repertoire of TRIM25 and highlights ubiquitin‐mediated control of core autophagy machinery as a critical checkpoint in antiviral defense [41].

Importantly, we demonstrate that ZNF33B promotes JEV replication by alleviating TRIM25‐mediated autophagy suppression. ZNF33B overexpression increased the levels of autophagy‐related proteins (ATG5, ATG7, Beclin1, LC3ǁ) and enhanced autophagic flux, while ZNF33B depletion had the opposite effect. Rescue experiments confirmed that ZNF33B reversed TRIM25‐induced downregulation of ATG7 and JEV NS3/NS5 proteins, and abrogated TRIM25‐mediated ATG7 ubiquitination. Furthermore, pharmacological inhibition of autophagy abolishes the proviral effect of ZNF33B, confirming that autophagy activation is essential for ZNF33B‐mediated JEV replication. Our study also hints at broader implications for flavivirus‐host interactions. Notably, the enhancement of ZIKV replication, but not CSFV, by ZNF33B suggests that this pathway may be selectively exploited by flaviviruses that depend on autophagy and m^6^A‐mediated host regulation, rather than representing a universal viral strategy [42]. In vivo studies using an AAV‐ZNF33B mouse model substantiate the physiological relevance of this pathway, showing that AAV‐mediated ZNF33B expression in mice increases global brain m^6^A levels, reduces TRIM25 expression, elevates ATG7 abundance, and exacerbates JEV neuropathology and mortality. These data establish the m^6^A‐TRIM25‐ATG7‐autophagy axis as a key regulatory pathway in JEV pathogenesis, integrating two well‐characterized host response pathways (epitranscription and autophagy) in a novel viral evasion strategy.

## 4 Conclusion

**FIGURE 10:**
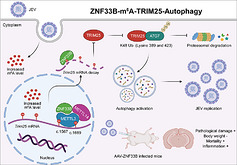
A model proposing that ZNF33B facilitates JEV replication by regulating the m^6^A‐TRIM25‐autophagy axis. Upon JEV infection, ZNF33B recruits METTL14 to stabilize the METTL3‐METTL14 m^6^A methyltransferase complex, leading to increased m^6^A modification of host transcripts, including *Trim25* mRNA. ZNF33B selectively binds m^6^A‐modified sites on *Trim25* mRNA and accelerates its decay, resulting in reduced TRIM25 protein abundance. TRIM25 normally restricts autophagy by catalyzing K48‐linked ubiquitination and proteasomal degradation of ATG7; thus, its suppression relieves autophagy inhibition and enhances autophagic flux, creating a cellular environment favorable for JEV replication.

Collectively, our study reveals a previously unrecognized mode of viral immune evasion in which an RNA‐binding zinc‐finger protein coordinates epitranscriptomic modification and ubiquitin‐mediated autophagy control to favor viral replication (Figure 10). By linking m^6^A‐dependent RNA decay to suppression of TRIM25 and activation of autophagy, we provide mechanistic insight into how flaviviruses rewire host regulatory networks at multiple levels. These findings nominate ZNF33B and components of the m^6^A‐TRIM25‐ATG7 axis as potential targets for host‐directed antiviral interventions and broaden the conceptual framework for understanding epitranscriptomic‐autophagic crosstalk in viral pathogenesis.

## 5 Experimental Section

### 5.1 Cell and Virus

The JEV P3 strain was a gift from Professor Yi‐ling Lin (Academia Sinica, Taiwan, China). ZIKV was a gift from Professor Wenchun Fan (Zhejiang University, Hangzhou, China). CSFV (Classical Swine Fever Virus) and JEV carrying a GFP reporter gene (JEV‐GFP) were preserved in our lab. All viruses are cultivated in baby hamster kidney (BHK‐21) cells. Swine kidney‐6 (SK6) cells, Porcine kidney‐15 (PK‐15) Cas9 cells and Human embryonic kidney HEK293T (HEK293T) cells were incubated in Dulbecco's modified Eagle's minimal essential medium (DMEM; Invitrogen, USA) supplemented with 10% fetal bovine serum (FBS; Hyclone), along with streptomycin sulfate (10 µg/mL, GENVIEW) and penicillin (100 U/mL, GENVIEW) at a consistent temperature of 37°C in a 5% CO_2_ environment.

### 5.2 Plasmids Construction and Transfection

pCAGGS‐YTHDF2‐HA was provided by Professor Hongbo Zhou (Huazhong Agricultural University). As described previously, cDNA encoding full‐length ZNF33B was cloned into pCMV‐C‐MYC, pCAGGS‐HA, and pTRIP‐3Flag‐RFP. The plasmids with epitope tags for other host genes: METTL3‐FLAG, METTL14‐FLAG, YTHDF1‐FLAG, YTHDF3‐FLAG, FTO‐FLAG, METTL3‐HA, METTL14‐HA, WTAP‐HA, FTO‐HA, ATG5‐MYC, ATG7‐MYC, TRIM25‐MYC, EGFP‐TRIM25, EGFP‐LC3, and mCherry‐GFP‐LC3. Constructs of TRIM25‐FLAG, TRIM25 ΔRING‐FLAG, and TRIM25 ΔSPRY‐FLAG were cloned into the pCR3.1‐FLAG vector with a C‐terminal FLAG‐tag fusion. The ATG7 mutants, including K389R, K423R, and K652R, were generated from the pCMV‐ATG7‐MYC plasmid through PCR‐based site‐directed mutagenesis. The TRIM25 m^6^A mutants (A to G) at positions c. 1492 bp, c. 1567 bp, and c. 1669 bp were generated from the TRIM25‐FLAG plasmid through site‐directed mutagenesis. Primers employed for the generation of mutants are presented in Table S1. Ub‐HA, K48 Ub‐HA, and K63 Ub‐HA were preserved in our laboratory. All constructs were confirmed by sequencing. Plasmid DNAs were transfected into cells using jetPRIME (polyplus, USA) or Lipofectamine 3000 (Invitrogen, USA).

### 5.3 Reagents and Antibodies

3‐Deazaadenosine (DAA, HY‐W013332), Actinomycin D (ActD, HY‐17559), Cycloheximide (CHX, HY‐12320), MG132 (HY‐13259), 3‐Methyladenine (3‐MA, HY‐19312), Chloroquine (CQ, HY‐17589A), Bafilomycin A1 (Baf.A1, HY‐100558), and DMSO (HY‐Y0320) were purchased from MCE (NY, USA). The antibodies used in the present study include JEV Envelope (GeneTex, Cat. # GTX125867), JEV NS3 (GeneTex, Cat. # GTX125868), ZIKV NS3(GeneTex, Cat. # GTX133309), JEV NS5 (GeneTex, Cat. # GTX131359), GAPDH (Proteintech, Cat. # 60004‐1‐Ig), MYC (Proteintech, Cat. # 16286‐1‐AP, 60003‐2‐Ig), DYKDDDDK (FLAG) (Proteintech, Cat. # 20543‐1‐AP), DYKDDDDK (FLAG) (MBL, Cat. # M185‐6), GFP (Proteintech, Cat. # 50430‐2‐AP, 66002‐1‐Ig), HA (Proteintech, Cat. # 51064‐2‐AP, 66006‐2‐Ig), IgG (Proteintech, Cat. # 30000‐0‐AP), m^6^A (Proteintech, Cat. # 68055‐1‐Ig), METTL3 (Proteintech, Cat. # 15073‐1‐AP), METTL14 (Proteintech, Cat. # 26158‐1‐AP), TRIM25 (Proteintech, Cat. # 12573‐1‐AP), ATG5 (Proteintech, Cat. # 10181‐1‐AP), ATG7 (Proteintech, Cat. # 10088‐2‐AP), ATG12 (Abclonal, Cat. # A22788), LC3B (Abclonal, Cat. # A19665), Beclin 1 (Abclonal, Cat. # A21191), SQSTM1/p62 (Abclonal, Cat. # A19700), HRP‐conjugated goat anti‐rabbit (MBL, Cat. # 458) IgG (H+L), and HRP‐conjugated goat anti‐mouse (MBL, Cat. # 330) secondary antibodies. The antibody against CSFV E2 was prepared in our lab. Alexa Fluor Plus 647 Goat anti‐rabbit IgG (H + L) highly cross‐adsorbed secondary antibody (Cat. # A32733), Alexa Fluor 488 goat anti‐mouse IgG (H + L) cross‐adsorbed secondary antibody (Cat. # A32731), and Alexa Fluor 568 goat anti‐rabbit IgG (H + L) cross‐adsorbed secondary antibody (Cat. # A11011) were obtained from Invitrogen (USA).

### 5.4 CRISPR‐Cas9‐Based Genome Editing

A guide RNA (gRNA) targeting the ZNF33B gene was designed and cloned into a linearized lenti‐sgRNA‐EGFP vector. Lentiviruses containing these sgRNAs were generated and then utilized to transduce PK15‐Cas9 cells. 3 days after transduction, EGFP‐positive cells were sorted and collected using fluorescence‐activated cell sorting (FACS). The identification of knockout cells was accomplished through a sequential process: monoclonal cells were lysed, genomic DNA was extracted, the target locus was amplified by means of gene‐specific primers, and the PCR products were validated through Sanger sequencing. The primer sequences employed in this study are presented in Table S2.

### 5.5 RNA Interference

siRNAs designed to specifically silence human METTL3, METTL14, and TRIM25 were produced by Tsingke (China) and delivered using Lipo3000 (Thermo, USA) according to the manufacturer's instructions. The detailed primers of these siRNAs are presented in Table S3.

### 5.6 Western Blotting

The cells were gently lysed using NP40 lysis buffer, which consisted of 1% NP40, 50 mM Tris‐HCl, 150 mM NaCl, and 1 mM EDTA. After that, the sample underwent centrifugation at a speed of 12 000 revolutions per minute (rpm) for 5 min. Following centrifugation, the supernatant was collected and thoroughly mixed with 5×loading buffer. Subsequently, the prepared samples were introduced into a 10% SDS‐PAGE gel. After the electrophoresis, the proteins were transferred from the gel to polyvinylidene fluoride (PVDF) membranes (Roche, UK) through a wet transfer method. Thereafter, the PVDF membranes were blocked and then incubated with specific antibodies to detect the target proteins. The image was developed using Bio‐Rad ChemiDoc XRS+ instrument and Image Lab software.

### 5.7 Immunoprecipitation

The protein supernatant was prepared and then incubated at 4°C with rotation. During the incubation, a proper amount of antibody, along with Protein A/G magnetic beads (Thermo, USA), was added. The incubation process continues for a duration ranging from 8 to 12 h. After the incubation period, the combined mixture underwent magnetic separation. Post‐separation, it was washed five times (10 min per time) using an iced wash buffer. The elution of proteins mixed with 2× loading buffer was heated at 95°C in the metal bath for 10 min. The obtained proteins were then put to use in an immunoblot examination.

### 5.8 Viral Titration

The cultured supernatant was serially diluted and introduced to BHK‐21 cell monolayers at a controlled temperature of 37°C for exactly 2 h. Subsequently, cells were gently overlaid with DMEM supplemented with 3% carboxymethyl cellulose (CMC) and 2% FBS. After an appropriate duration of incubation, the cells were fixed with 4% paraformaldehyde and stained with 1% crystal violet dye. Then the viral plaques were counted for in‐depth analysis.

### 5.9 Confocal Microscopy

After the suitable administration, the cells on the confocal culture dish underwent a series of procedures. First, cells were fixed using 4% paraformaldehyde for a duration of 20 min. Next, the cells were made permeable with 0.2% Triton X‐100, and then blocked with 5% bovine serum albumin (BSA). Following these steps, the cells were incubated with primary antibodies. After that, they were exposed to fluorescent‐dye‐conjugated secondary antibodies. To stain the nuclei, DAPI (Sigma‐Aldrich, USA) was employed. Finally, the visualization of images was conducted using the Zesis LSM800 microscope system (Zesis, Germany).

### 5.10 Quantitative PC

Total RNA isolated by the TRIzol reagent (Invitrogen, USA) was reverse transcribed into cDNA with the Hifair AdvanceFast first Strand cDNA Synthesis Kit (Yeasen, China), adhering to the manufacturer's instructions. For the quantitative real‐time PCR analysis, the CFX Opus Real‐time PCR system (Bio‐Rad) was employed, along with the SYBR qPCR Master Mix (Vazyme, China). The target primers utilized in this process are presented in Table S4. To determine the relative mRNA expression accurately, the values were normalized to GAPDH.

### 5.11 Analysis of mRNA Half‐Life Using ActD

Briefly, Actinomycin D (ActD) (5 µg/mL, MCE, USA) was utilized to impede the mRNA synthesis. After that, cells were collected at the pre‐set treatment durations for qPCR quantification. The non‐linear regression curve fitting was employed to capture the decay dynamics of the mRNA.

### 5.12 Analysis of Protein Half‐Life Using CHX

Under introduced with the corresponding plasmids over a period of 24 h, cells were treated with CHX (50 µg/ml) for the indicated duration. The Western blot analysis was performed to accurately determine the levels of JEV NS3 and NS5 expression.

### 5.13 RNA‐Fluorescence In Situ Hybridization

The specified cells were fixed in confocal dishes using 4% paraformaldehyde, followed by permeabilization in 70% ethanol overnight at 4°C. In strict accordance with the manufacturer's instructions (RNASweAMI in situ Hybridization Protocol, Servicebio), RNA in situ hybridization was performed following the RNASweAMI protocol, using a swine *Trim25*‐targeting probe designed and produced by Servicebio (Table S5). After the in situ hybridization was completed, the dishes were washed twice with 1× SSC and subjected to immunostaining. Subsequently, staining for anti‐FLAG‐ZNF33B was performed, followed by DAPI counterstaining. The image was performed using a Zeiss LSM880 microscope system.

### 5.14 RNA Immunoprecipitation

Briefly, a portion of the cell lysate was reserved as the input. Meanwhile, the rest of the lysates were divided into aliquots and incubated overnight at 4°C with gentle rotation. During the overnight incubation, lysates were combined with either anti‐HA antibodies or control IgG antibodies that had been pre‐encapsulated onto Protein A/G magnetic beads (Thermo, USA). Subsequently, the immunoprecipitated pellets underwent NT2 buffer wash and proteinase K treatment (Thermo, USA) at 55°C for 20 min. Then, the RNA was purified and subjected to qPCR quantification. RNA used in RIP‐seq was first rRNA‐depleted, followed by library construction using the NEBNext Ultra II Direct RNA Library preparation Kit with 10 cycles of PCR amplification and sequenced on an Illumina NovaSeq 6000 platform with 150 bp paired‐end reads. RIP‐seq was performed in Seqhealth (Wuhan, China).

### 5.15 RNA Sequencing

After the extraction of the RNA from SK6 cells using TRIzol Reagent, DNA digestion was performed by DNase I. The quality of the RNA was verified by the A260/A280 ratio using the Nanodrop instrument. The integrity of the RNA was further confirmed through 1.5% agarose gel electrophoresis. Ultimately, the qualified RNAs were measured using the Qubit 3.0 device along with the Qubit RNA Broad Range Assay kit (Life Technologies, Q10210). For the preparation of the stranded RNA sequencing library, 2 µg of the total RNA was employed for Illumina using the KCTM Stranded mRNA Library Prep Kit (Wuhan Seqhealth Co., Ltd., China, DR08402) in strict accordance with the manufacturer's instructions. Subsequently, the PCR products that range from 200 to 500 base pairs (bps) were enriched and quantified. Finally, these products were sequenced on the Novaseq 6000 sequencer (Illumina) with the PE150 model to obtain the necessary sequencing data for further analysis.

### 5.16 RIP‐Seq Analysis

After obtaining the sequencing raw data, the raw data is first filtered to obtain high‐quality sequencing data (clean data). The sequencing data (clean data) is aligned to the swine genome to obtain comprehensive transcript information, and gene expression quantification, as well as GO and KEGG Pathway analyses are performed. Meanwhile, genome‐wide de novo peak calling is conducted on the alignment results to study the binding preference of proteins on RNA, and motif analysis is performed on the binding sites.

### 5.17 M^6^A‐IP‐qPCR (MeRIP‐qPCR)

Isolated mRNA was fragmented into ∼300 nt fragments using RNA Fragmentation Reagents (Vazyme, catalog number: N402‐01). Five percent of the fragmented mRNAs were kept as input. MeRIP was performed using anti‐m^6^A antibody (Synaptic Systems, 202003), essentially as detailed in the RIP procedure. IgG (Santa Cruz Biotechnology, catalog number: sc‐2025) was used as a control. The m^6^A enrichment was measured using qPCR.

### 5.18 M^6^A Dot Blot Assay

Total RNA was isolated using Trizol (Invitrogen) according to the manufacturer's instructions. mRNA purification was performed in two rounds using the Dynabeads mRNA Purification kit (Thermofisher Scientific). The purified mRNA was denatured at 55°C for 30 min, and then 1.5 µL of the sample was directly applied to the Hybond‐N^+^ membrane. After crosslinking using the UV crosslinker, the membrane was blocked, and then probed with anti‐m^6^A antibody and HRP‐conjugated Goat anti‐mouse IgG. After exposure, the membrane was stained with 0.02% methylene blue dye buffer. The signal density of the dot‐blot experiment was quantified by ImageJ software (Media Cybernetics).

### 5.19 Ubiquitination Assay

Cells transiently transfected with ATG7‐MYC and Ub‐HA plasmids for 36 h were lysed with NP40 buffer. The resulting lysates for immunoprecipitation were treated with specific antibodies along with Protein A+G magnetic agarose (Beyotime, China). Subsequently, the beads underwent a sequential washing process with NP40 buffer. The immunoprecipitants were then eluted, and the samples, combined with 2× loading buffer, were denatured at 95°C for a duration of 10 min. The target proteins and their corresponding ubiquitinated form were separated through SDS‐PAGE and detected by immunoblotting.

### 5.20 Mice

Balb/c mice (6–7 weeks old, 18–20 g) were obtained from the Laboratory Animal Centre of Huazhong Agriculture University (Wuhan, China) and housed in a specific pathogen‐free (SPF) facility with controlled environmental conditions (temperature: 25 ± 1°C; relative humidity: 40%–60%). Animals were maintained under a 12‐h light/dark cycle with ad libitum access to autoclaved food and water. The animal experiments were independently repeated twice with similar results, and data from one representative experiment (n = 5 per group) are presented. All experimental protocols were approved by the Scientific Ethics Committee of Huazhong Agricultural University (Approval No. HZAUMO‐2026‐0019) and conducted in accordance with ARRIVE guidelines.

### 5.21 Adeno‐Associated Virus Injection

ZNF33B overexpression was achieved via an adeno‐associated virus (AAV) vector delivery system. Briefly, the negative control vector and ZNF33B‐expressing AAV vector underwent synthesis and subcloning into the AAV‐1205 vector (pAAV‐CAG‐ZNF33B‐3×FLAG‐P2A‐EGFP, TSINGKE Biotechnology). The recombinant constructs were verified by DNA sequencing. Subsequently, AAV‐ZNF33B and negative control viral particles, with a dosage of 1.5 × 10^13^ v.g. per mouse, were administered via the caudal vein to 7‐week‐old Balb/c mice using a microsyringe pump.

### 5.22 Virus Infection In Vivo

Mice that had been administered with AAV‐EV or AAV‐ZNF33B at 7 weeks of age were, after 35 days of AAV expression (i.e., at 12 weeks of age), injected intraperitoneally (i.p.) with the JEV P3 strain (1 × 10^6^ PFU). After 8 days, the mouse brains were sampled for analysis by western blot, ELISA, hematoxylin and eosin (H&E) staining, or immunohistochemistry (IHC) assay. Mouse survival and body weight were recorded daily for 8 days.

### 5.23 Enzyme‐Linked Immunosorbent Assay

The brain was mechanically homogenized in phosphate‐buffered saline (PBS) containing Halt Protease and Phosphatase Inhibitor Cocktail (Thermo, cat#78442). The mixture was then centrifuged at 12 000 × g for 10 min at 4°C, after which the supernatants were collected. The concentrations of IL‐1β, IL‐6, TNF‐α, and m6A were measured using ELISA kits from MMBIO (China), in strict accordance with the manufacturer's instructions. The final concentration was determined through an enzyme‐linked immunosorbent assay (SPARK10M, TECAN) with the optical density (OD) value measured at 450 nm.

### 5.24 Brain Histology

Brains from JEV‐infected mice were fixed in 4% formaldehyde solution and then embedded in paraffin. Paraffin sections were stained with hematoxylin‐eosin solution and then observed by light microscopy for histological changes.

For immunofluorescence staining based on TSA (tyramide signal amplification) staining technology, brain tissues were stained using antibodies against JEV NS3, TRIM25, m6A, and ATG7. Nuclei were stained with DAPI.

### 5.25 Statistical Analysis

The data are presented as mean ± standard deviation (SD). Statistical comparisons between two groups were performed using two‑tailed unpaired Student's *t*‑test. For comparisons among three or more groups, one‑way or two‑way analysis of variance (ANOVA) was used, followed by Tukey's or Šídák's post‑hoc test as appropriate. The survival was determined with the Kaplan–Meier method. All statistical analyses were performed using GraphPad Prism 9 software.

## Author Contributions

J.D. performed the experiment, analyzed the data, and drafted the manuscript. C.L., J.L., J.Z., H.Z., and S.W. helped with the investigation. H.C. provided the materials. H.X., X.L., and Q.P. contributed to the experimental design, manuscript review and editing, provision of resources, acquisition of funding, and project supervision.

## Conflicts of Interest

The authors declare no competing interests.

## Supporting information




**Supporting File 1**: advs76122‐sup‐0001‐SuppMat.docx

**Supporting File 2**: advs76122‐sup‐0002‐DataFile.xlsx

## Data Availability

The RIP‐seq and RNA‐seq data generated in this study have been deposited in the NCBI Sequence Read Archive (SRA) database under accession numbers PRJNA1471351 and PRJNA1471055. The primary data are also available from the corresponding author upon request.
