# Supplementary material for: ZNF33B Promotes Japanese Encephalitis Virus Infection by Regulating the Stability of M6A‐Modified Trim25 to Control the Autophagy Process
Source: Adv Sci (Weinh). 2026 Jun 18;13(51):e76122. doi: 10.1002/advs.76122 (PMC13336372; doi:10.1002/advs.76122)
Supplement: Supplementary file 1 [file ADVS-13-e76122-s001.docx]

**Supplementary information**

ZNF33B promotes Japanese encephalitis virus infection by regulating the stability of m^6^A-modified *Trim25* to control the autophagy process

Jian Du, Chunwei Li, Jiyuan Luo, Huizhi Zhang, Jinyan Zhang, Suya Wang, Huanchun Chen, Hongli Xu, Xiangmin Li, Ping Qian

Corresponding: Ping Qian

Email: [qianp@mail.hzau.edu.cn](mailto:qianp@mail.hzau.edu.cn)

**This file includes:**

Figures S1 to S11

Tables S1 to S6

**Figure S1**

**Fig S1.** **JEV infection enhanced the m^6^A level.**

**(A)** The confocal microscope observation of the m^6^A signal in HEK293T cells infected by JEV for 36 and 48 h. Scale bar: 5 μm.

**(B)** The levels of m^6^A and JEV NS5 protein were quantitatively assessed by measuring the fluorescence intensity with the "ImageJ" software.

**(C)** Immunoblot analysis of lysates from HEK293T cells transfected with METTL3-FLAG, followed by Mock or JEV infection for 48 h. The expression of METTL3 was assessed by measuring the band grayscale with the "ImageJ" software.

**(D)** Immunoblot analysis of lysates from HEK293T cells transfected with METTL14-FLAG, followed by Mock or JEV infection for 48 h. The expression of METTL14 was assessed by measuring the band grayscale with the "ImageJ" software.

**(E)** Immunoblot analysis of lysates from Mock or JEV-infected HEK293T cells for 48 h. The expression of METTL3 and METTL14 was assessed by measuring the band grayscale with the "ImageJ" software.

All experiments were conducted in triplicate, and data are represented as mean ± SD. Statistical analysis was performed by a two-tailed Student's *t*-test (B, right panel)or one-way ANOVA with Tukey’s multiple comparisons (B, left panel).

**Figure S2**

**Fig S2. ZNF33B facilitates JEV infection in an m6A-dependent manner.**

**(A)** The mRNA level of *Mettl3* gene was assessed by qPCR in HEK293T cells transfected with NC or METTL3 siRNAs.

**(B)** Immunoblot analysis of the expression of JEV NS3 and NS5 in HEK293T cells transfected with ZNF33B-FLAG and METTL3 siRNA, followed by JEV infection for 36 h. The expressions of JEV NS3 and NS5 were assessed by measuring the band grayscale with the "ImageJ" software.

**(C)** Immunoblot analysis of the expression of JEV NS3 and NS5 in HEK293T cells transfected with ZNF33B-FLAG and FTO-HA, followed by JEV infection for 36 h. The expressions of JEV NS3 and NS5 were assessed by measuring the band grayscale with the "ImageJ" software.

All experiments were conducted in triplicate, and data are represented as mean ± SD. Statistical analysis was performed by a two-tailed Student's *t*-test (A).

**Figure S3**

**Fig S3.** **The interaction between ZNF33B and m^6^A proteins.**

**(A)** Immunoblot analysis of the association of ZNF33B with METTL3 by immunoprecipitation of lysates from HEK293T cells co-transfected with ZNF33B-HA and METTL3-FLAG. The cell lysates were immunoprecipitated with anti-FLAG antibody.

**(B)** Immunoblot analysis of the association of ZNF33B with METTL14 by immunoprecipitation of lysates from HEK293T cells co-transfected with ZNF33B-HA and METTL14-FLAG. The cell lysates were immunoprecipitated with anti-FLAG antibody.

**(C)** Immunoblot analysis of the association of ZNF33B with WTAP by immunoprecipitation of lysates from HEK293T cells co-transfected with ZNF33B-FLAG and WTAP-HA. The cell lysates were immunoprecipitated with anti-FLAG antibody.

**(D)** Immunoblot analysis of the association of ZNF33B with YTHDF1 by immunoprecipitation of lysates from HEK293T cells co-transfected with ZNF33B-HA and YTHDF1-FLAG. The cell lysates were immunoprecipitated with anti-FLAG antibody.

**(E)** Immunoblot analysis of the association of ZNF33B with YTHDF2 by immunoprecipitation of lysates from HEK293T cells co-transfected with ZNF33B-HA and YTHDF2-FLAG. The cell lysates were immunoprecipitated with anti-FLAG antibody.

**(F)** Immunoblot analysis of the association of ZNF33B with YTHDF3 by immunoprecipitation of lysates from HEK293T cells co-transfected with ZNF33B-HA and YTHDF3-FLAG. The cell lysates were immunoprecipitated with anti-FLAG antibody.

**(G)** Immunoblot analysis of the association of ZNF33B with FTO by immunoprecipitation of lysates from HEK293T cells co-transfected with ZNF33B-HA and FTO-FLAG. The cell lysates were immunoprecipitated with anti-FLAG antibody.

**(G)** Immunoblot analysis of the association of ZNF33B with m6A-related proteins by immunoprecipitation of lysates from HEK293T cells co-transfected with ZNF33B-HA. The cell lysates were immunoprecipitated with anti-HA antibody.

**Figure S4**

**Fig S4.** **Analysis of RIP-seq and RNA-seq.**

**(A)** A volcano plot illustrating the differentially expressed genes (DEGs), with upregulated genes in the JEV-infected SK6 cells represented in red and downregulated genes in blue, compared to mock-infected SK6 cells.

**(B and C)** Gene Ontology (GO) and Kyoto Encyclopedia of Genes and Genomes (KEGG) analyses of genes with differential RIP peaks.

**(D)** A volcano plot illustrating analysis of mRNA alternative splicing events, with SE event represented in orange, A3SS event represented in red, A5SS event represented in blue, MXE event represented in green, and RI event represented in purple.

**(E and F)** GO and KEGG analyses of mRNA alternative splicing events.

**Figure S5**

**Fig S5. The validation of ZNF33B-associated mRNAs.**

**(A)** RIP assay using anti-FLAG antibody and qPCR analysis of the association between ZNF33B protein and *Dhx58*, *Trim25*, *Znfx1*, and *Coro1a* mRNAs with specific primers in SK6 cells transfected with FLAG-tagged ZNF33B, followed by JEV infection for 48 h. The cell lysates were immunoprecipitated with anti-FLAG antibodies.

**(B)** Immunoblot analysis of lysates from SK6 cells transfected with ZNF33B-FLAG, followed by JEV infection for 48 h.

**(C)** qPCR analysis of the levels of *Dhx58*, *Trim25*, *Znfx1*, and *Coro1a* mRNAs in SK6 cells transfected with ZNF33B-FLAG, followed by JEV infection for 48 h.

All experiments were conducted in triplicate, and data are represented as mean ± SD. Statistical analysis was performed by a two-tailed Student's *t*-test (A and C).

**Figure S6**

**Fig S6.** **IGV tracks displaying the indicated ZNF33B’ binding sites on *Dhx58* (A), *Znfx1* (B), and *Coro1a* (C) mRNAs from the RIP-seq data.**

**Figure S7**

**Fig S7.** **The effect of m^6^A writer proteins on the level of TRIM25.**

**(A)** Immunoblot analysis of lysates from HEK293T cells transfected with METTL3-FLAG and TRIM25-MYC. The expression of TRIM25 was assessed by measuring the band grayscale with the "ImageJ" software.

**(B)** Immunoblot analysis of lysates from HEK293T cells transfected with METTL14-FLAG and TRIM25-MYC. The expression of TRIM25 was assessed by measuring the band grayscale with the "ImageJ" software.

**(C)** The m^6^A modification site of *Trim25* mRNA was predicted by SRAMP ([www.cuilab.cn/m6asiteapp](http://www.cuilab.cn/m6asiteapp)).

**Figure S8**

**Fig S8.**  **TRIM25 deficiency promotes JEV replication by antagonizing autophagy.**

**(A)** Immunoblot analysis of lysates from HEK293T cells transfected with TRIM25 siRNA. The expressions of JEV E, NS3, and NS5 were assessed by measuring the band grayscale with the "ImageJ" software.

**(B)** Immunoblot analysis of lysates from HEK293T cells transfected with TRIM25 siRNA, with or without Baf.A1 treatment (20 nM). The expressions of MAP1LC3B and SQSTM1/p62 were assessed by measuring the band grayscale with the "ImageJ" software.

**(C)** The statistical analysis of MAP1LC3B and SQSTM1/p62 expressions by GraphPad Prism 9 software.

All experiments were conducted in triplicate, and data are represented as mean ± SD. Statistical analysis was performed by a two-tailed Student's *t*-test (C).

**Figure S9**

**Fig S9. The effect of TRIM25 on endogenous ATG7 ubiquitination.**

**(A)** Immunoblot analysis of the effect of TRIM25 on ATG7 ubiquitination from HEK293T cells transfected with TRIM25-FLAG, followed by MG132 (10 μM) treatment for 6 h. The lysates were subjected to precipitation using anti-ATG7 antibodies for the enrichment of ubiquitinated proteins, followed by probing with the specified antibodies.

**(B)** Immunoblot analysis of the effect of TRIM25 on ATG7 ubiquitination from HEK293T cells transfected with TRIM25 siRNA, followed by MG132 (10 μM) treatment for 6 h. The lysates were subjected to precipitation using anti-ATG7 antibodies for the enrichment of ubiquitinated proteins, followed by probing with the specified antibodies.

**(C)** Immunoblot analysis of the effect of TRIM25 on ATG7 ubiquitination from HEK293T cells co-transfected with TRIM25-FLAG and K48 Ubi-HA, followed by MG132 (10 μM) treatment for 6 h. The lysates were subjected to precipitation using anti-ATG7 antibodies for the enrichment of ubiquitinated proteins, followed by probing with the specified antibodies.

**(D)** Immunoblot analysis of the effect of TRIM25 on ATG7 ubiquitination from HEK293T cells co-transfected with TRIM25-FLAG and K63 Ubi-HA, followed by MG132 (10 μM) treatment for 6 h. The lysates were subjected to precipitation using anti-ATG7 antibodies for the enrichment of ubiquitinated proteins, followed by probing with the specified antibodies.

**Figure S10**

**Fig S10. The effect of ZNF33B on other *Flaviviridae* virus replication.**

**(A)** Immunoblot analysis of lysates from SK6 cells transfected with ZNF33B-FLAG, followed by ZIKV infection for 60 h. The expression of ZIKV NS3 proteins was assessed by measuring the band grayscale with the "ImageJ" software.

**(B)** The observation of ZIKV NS3 in ZIKV-infected SK6 cells by IFA. Scale bar, 10 μm.

**(C)** Immunoblot analysis of the expression of ZIKV NS3 in ZIKV-infected PK-15 WT and ZNF33B KO cells. The expression of ZIKV NS3 was assessed by measuring the band grayscale with the "ImageJ" software.

**(D)** The observation on ZIKV NS3 in ZIKV-infected PK-15 Cas9 and ZNF33B KO cells by IFA. Scale bar, 10 μm.

**(E)** Immunoblot analysis of lysates from SK6 cells transfected with ZNF33B-FLAG, followed by CSFV infection for 60 h. The expression of CSFV E2 proteins was assessed by measuring the band grayscale with the "ImageJ" software.

**(F)** The observation of CSFV E2 in CSFV-infected SK6 cells by IFA. Scale bar, 10 μm.

**(G)** Immunoblot analysis of the expression of CSFV E2 in CSFV-infected PK-15 WT and ZNF33B KO cells. The expression of CSFV E2 was assessed by measuring the band grayscale with the "ImageJ" software.

**(H)** The observation on CSFV E2 in CSFV-infected PK-15 Cas9 and ZNF33B KO cells by IFA. Scale bar, 10 μm.

All experiments were conducted in triplicate, and data are represented as mean ± SD. Statistical analysis was performed by a two-tailed Student's *t*-test (A, C, E, and G).

**Figure S11**

**Fig S11. The detection of the m6A level.**

The levels of m6A in brain-specific ZNF33B-overexpressing mice infected with JEV P3 strain were detected by ELISA.

All experiments were conducted in triplicate, and data are represented as mean ± SD. Statistical analysis was performed by a two-tailed Student's *t*-test.

**Table S1 Primers employed for the generation of mutants**

| **Mutants** | **Sequences (5’-3’)** |
| --- | --- |
| TRIM25 MUT1-F | catcctggTctacaacaccgcccacaacaaggtg |
| TRIM25 MUT1-R | ggtgttgtagAccaggatgactttaacggcatac |
| TRIM25 MUT2-F | tcccaggTctaccagccacatccccagaggttcaccta |
| TRIM25 MUT2-R | atgtggctggtagAcctgggacgtgtcggccgcaga |
| TRIM25 MUT3-F | ctgcagaagaTcaacttctgcggggtaggcgtctgc |
| TRIM25 MUT3-R | agaagttgAtcttctgcagctccacctcccagtag |
| ATG7 K389R-F | gtggacaatgccaggatctcctattccaaccctgtgaggc |
| ATG7 K389R-R | ggagatcctggcattgtccacaaatgtgatgtgtctgacgc |
| ATG7 K423R-F | gaccgcctccagagaatattccccggagtgaatgcc |
| ATG7 K423R-R | attctctggaggcggtctgctgctgccagggc |
| ATG7 K652R-F | gcttgttcttccagagttcttgatcaatatgaacgggaagg |
| ATG7 K652R-R | caagaactctggaagaacaagctgtacatttgtcaaatgcc |

**Table S2 sgRNA employed for the gene knockout**

| **Genes** | **Sequences (5’-3’)** |
| --- | --- |
| Swine ZNF33B | AGTCCCTAGGAATAGTAAAC |

**Table S3 siRNAs used for RNA interference**

| **Genes** | | **Sequences (5’-3’)** |
| --- | --- | --- |
| Human *Mettl3* | Sense #1 | CUACAGAUCCUGAGUUAGA(dT)(dT) |
|  | Antisense #1 | UCUAACUCAGGAUCUGUAG(dT)(dT) |
|  | Sense #2 | GGAACAAUCCAUUGUUGAA(dT)(dT) |
|  | Antisense #2 | UUCAACAAUGGAUUGUUCC(dT)(dT) |
|  | Sense #3 | CAAGAUUGAGUUAUUUGGA(dT)(dT) |
|  | Antisense #3 | UCCAAAUAACUCAAUCUUG(dT)(dT) |
| Human *Mettl14* | Sense #1 | GGAUGAGUUAAUAGCUAAA(dT)(dT) |
|  | Antisense #1 | UUUAGCUAUUAACUCAUCC(dT)(dT) |
|  | Sense #2 | GCAUUGGUGCCGUGUUAAA(dT)(dT) |
|  | Antisense #2 | UUUAACACGGCACCAAUGC(dT)(dT) |
|  | Sense #3 | GGACAUAGACCUCAGAAUU(dT)(dT) |
|  | Antisense #3 | AAUUCUGAGGUCUAUGUCC(dT)(dT) |
| Human *Trim25* | Sense #1 | GGUGGAGCAGCUACAACAA(dT)(dT) |
|  | Antisense #1 | UUGUUGUAGCUGCUCCACC(dT)(dT) |
|  | Sense #2 | GCUCUGUCAGAGUGCUAUA(dT)(dT) |
|  | Antisense #2 | UAUAGCACUCUGACAGAGC(dT)(dT) |
|  | Sense #3 | GCAAAUGUUCCCAGCACAA(dT)(dT) |
|  | Antisense #3 | UUGUGCUGGGAACAUUUGC(dT)(dT) |

**Table S4 Primers for qPCR**

| **Gene** | **Sequences (5’-3’)** |
| --- | --- |
| Swine *Dhx58*-F | TGGTCCTTAGTCCTCTGCCTC |
| Swine *Dhx58*-R | TGGCTCCATCCACAGTCTCC |
| Swine *Trim25*-F | GAGGCCAACTCGACTCGAAA |
| Swine *Trim25*-R | CAGGCTTCGTGGTGATTCCT |
| Swine *Znfx1*-F | GACCTCCTGGAACAGGCAAA |
| Swine *Znfx1*-R | CTTCCACCTACCCGCACAAT |
| Swine *Coro1a*-F | CTTCCCCACACACAGGTTCCTC |
| Swine *Coro1a*-R | CCTGTGCTGCCCACTCAAGA |
| Swine *Gapdh*-F | GAGTGAACGGATTTGGCCG |
| Swine *Gapdh*-R | CACCCCATTTGATGTTGGCG |
| Human *Trim25*-F | AGAGCCTGACCAAGAGGGAT |
| Human *Trim25*-R | GTGGATTTGTGTGTGGACGC |
| Human *Mettl3*-F | TGGGGGTATGAACGGGTAGA |
| Human *Mettl3*-R | TGGTTGAAGCCTTGGGGATT |
| Human *Gapdh*-F | CATGGCCTTCCGTGTTCCTA |
| Human *Gapdh*-R | CCTGCTTCACCACCTTCTTG |

**Table S5 Probes for FISH**

| Probe name | Sequences (5’-3’) |
| --- | --- |
| Probe-*Trim25*-1 | GCTCAGGGCAGAAGAAGTCTCGAA |
| Probe-*Trim25*-2 | CGCCCTTTATCAGCTCATGGTTCA |
| Probe-*Trim25*-3 | AACACCTGCGAGCAGTAGGTGAAC |
| Probe-*Trim25*-4 | CCGAGATCTTGGTGTTGAACCACT |
| Probe-*Trim25*-5 | ATGAGGCAGTGGCAGGTTTCTTGG |

**Table S6 The daily** **body weight of mice**

|  |  | Body weight (g) | | | | | | | |
| --- | --- | --- | --- | --- | --- | --- | --- | --- | --- |
|  | | DAY1 | DAY 2 | DAY3 | DAY4 | DAY5 | DAY6 | DAY7 | DAY8 |
| EV | A1 | 26.40 | 24.81 | 25.71 | 25.13 | 23.92 | 21.29 | dead | dead |
|  | A2 | 27.15 | 25.83 | 26.74 | 26.41 | 24.47 | dead | dead | dead |
|  | A3 | 27.90 | 26.42 | 27.65 | 27.73 | 27.21 | 24.75 | 22.69 | dead |
|  | A4 | 25.50 | 23.39 | 24.62 | 23.99 | 22.97 | dead | dead | dead |
|  | A5 | 27.02 | 26.18 | 27.63 | 26.55 | 25.03 | 22.29 | dead | dead |
| ZNF33B | A6 | 24.25 | 23.64 | 24.48 | 23.42 | 21.83 | dead | dead | dead |
|  | A7 | 27.30 | 26.28 | 27.37 | 26.87 | dead | dead | dead | dead |
|  | A8 | 25.62 | 24.21 | 26.34 | 25.86 | 22.01 | 20.48 | dead | dead |
|  | A9 | 25.83 | 24.18 | 24.52 | 24.81 | 23.27 | dead | dead | dead |
|  | A10 | 27.56 | 26.11 | 27.06 | 26.97 | 24.47 | dead | dead | dead |
